# Educational attainment, electroencephalographic rhythms, cortical structure, and cognitive performance over 2 years in older adults with subjective memory complaints and brain amyloidosis

**DOI:** 10.1002/alz.70438

**Published:** 2025-07-14

**Authors:** Susanna Lopez, Harald Hampel, Claudio Del Percio, Giuseppe Noce, Roberta Lizio, Stefan J. Teipel, Martin Dyrba, Gabriel González‐Escamilla, Hovagim Bakardjian, Patrizia Andrea Chiesa, Enrica Cavedo, Andrea Vergallo, Pablo Lemercier, Giuseppe Spinelli, Michel J. Grothe, Marie‐Claude Potier, Fabrizio Stocchi, Chiara Coletti, Raffaele Ferri, Matteo Pardini, Marie‐Odile Habert, Simone Marziali, Bruno Dubois, Claudio Babiloni, and INSIGHT‐preAD study group

**Affiliations:** ^1^ Department of Physiology and Pharmacology “Erspamer” Sapienza University of Rome Rome Italy; ^2^ Alzheimer Precision Medicine (APM) AP‐HP Pitié‐Salpêtrière Hospital Sorbonne Université Paris France; ^3^ IRCCS Synlab SDN Naples Italy; ^4^ Oasi Research Institute‐IRCCS Troina Italy; ^5^ Department of Psychosomatic Medicine University of Rostock Rostock Germany; ^6^ German Center for Neurodegenerative Diseases (DZNE)–Gehlsheimer Str. Rostock Germany; ^7^ Department of Neurology Saarland University Homburg Germany; ^8^ Department of Neurology Institute of Memory and Alzheimer's Disease (IM2A) Pitié‐Salpêtrière Hospital Paris France; ^9^ Institut du Cerveau et de la Moelle épinière ICM, INSERM U1127 CNRS UMR 7225 Sorbonne Université Paris France; ^10^ Centre pour l'Acquisition et le Traitement des Images (CATI platform) Pitié‐Salpêtrière Hospital, Boulevard del'hôpital Paris France; ^11^ Laboratoire d'Imagerie Biomédicale CNRS INSERM Sorbonne Université Paris France; ^12^ IRCCS San Raffaele Rome Italy; ^13^ San Raffaele Telematic University Rome Italy; ^14^ Dipartimento di Neuroscienze Oftalmologia Genetica Riabilitazione e Scienze Materno‐infantili (DiNOGMI) Università di Genova Genova Italy; ^15^ Neurofisiopatologia IRCCS Ospedale Policlinico San Martino Genova Italy; ^16^ Department of Nuclear Medicine AP‐HP Pitié‐Salpêtrière Hospital Paris France; ^17^ Hospital San Raffaele Cassino Cassino Italy

**Keywords:** educational attainment, insight‐pre‐AD study, preclinical Alzheimer's disease (AD) amyloid‐b, resting‐state electroencephalographic (rsEEG) alpha rhythms, structural magnetic resonance imaging (sMRI), subjective memory complaint (SMC)

## Abstract

**INTRODUCTION:**

We investigated whether older adults with subjective memory complaints (SMC) and amyloid‐β accumulation may show clinical progression over 2 years, as measured by resting‐state electroencephalographic (rsEEG), structural magnetic resonance imaging (sMRI), and cognitive variables, depending on educational attainment.

**METHODS:**

We analyzed these markers in 84 SMC participants from INSIGHT‐Pre‐AD study, grouped by amyloid‐β deposition (^18^F‐florbetapir positron emission tomography) and educational attainment.

**RESULTS:**

In amyloid‐negative individuals, higher educational attainment was linked to greater posterior rsEEG alpha activity, possibly reflecting neuroprotective effects. Conversely, amyloid‐positive individuals with higher educational attainment showed reduced posterior rsEEG alpha rhythms and lower parietal cortical thickness, potentially indicating compensatory mechanisms counteracting early amyloidosis and neurodegeneration. No longitudinal changes were found in either group over 2 years.

**DISCUSSION:**

Education had a stable influence on rsEEG, sMRI, and cognitive markers over 2 years in SMC individuals. Longer follow‐up periods should be used to monitor brain status with those markers.

**Highlights:**

- Education, subjective memory complaint (SMC), and brain amyloid‐β deposition.
- Stable influence of education on resting‐state electroencephalographic (rsEEG), structural magnetic resonance imaging (sMRI), and cognitive markers over 2 years.
- Compensatory mechanism of education against early amyloidosis and neurodegeneration.
- Longer follow‐up periods to monitor brain status in SMC older adults with those markers.

## 1 BACKGROUND

Alzheimer's disease (AD) is the most prevalent aging‐related neurodegenerative disease belonging to dementia, so its early detection and monitoring, from subjective memory complaints (SMC) to mild cognitive impairment (ADMCI), are important research priorities. Indeed, AD progression is highly individualized and depends on several genetic, neurobiological, neurophysiological, psychosocial, socioeconomic, and environmental factors that prevent or counteract the neurodegenerative process, modulating a kind of brain/cognitive reserve (CR) and resilience.^1^ Such a reserve refers to the brain's ability to cope with neurodegeneration and maintain cognitive function, partially depending on lifestyle in association with high levels of educational attainment and related intellectual activities in work, cultural, and creative/leisure pursuits.^1^ The CR physiological basis includes enhanced neurogenesis, synaptic plasticity, neuromodulatory efficiency, and flexible reorganization of cerebral functional connectivity to compensate for neuropathological and neurodegenerative processes.^2^, ^3^, ^4^, ^5^, ^6^ Educational attainment is an important and convenient CR aspect because it reflects socioeconomic inequalities,^7^, ^8^, ^9^, ^10^ significantly influences brain health,^10^, ^11^, ^12^ and reduces the risk of developing dementia.^13^

CR was initially introduced to justify cognitive performance in patients with AD dementia (ADD) despite significant brain neuropathology observed by autoptic histological analyses^14^ and in vivo AD biomarkers.^3^, ^7^, ^15^ While in healthy aging, CR neuroprotective mechanisms can explain why older adults with higher educational attainment (Edu+) show delayed onset of cognitive deficits compared to those with lower educational attainment (Edu‐), CR compensatory mechanisms can support why along neurodegeneration those with Edu+ present resilient cognitive functions despite substantial brain atrophy and hypometabolism, as shown by magnetic resonance imaging (MRI) and positron emission tomography (PET) metabolic biomarkers.^3^, ^7^, ^16^, ^17^, ^18^, ^19^, ^20^, ^21^, ^22^, ^23^, ^24^, ^25^ These compensatory mechanisms can indeed explain the results on clinical‐cognitive status and autoptic neuropathological burden (e.g., neocortical and hippocampal amyloid‐β (Aβ)‐composed neuritic plaques, neocortical cerebral amyloid angiopathy, Braak stages for tau) obtained in hundreds of elderly patients with dementia.^26^

AD and CR impact the brain's neurophysiological thalamocortical oscillatory mechanisms that regulate cortical arousal and vigilance during wakefulness, as reflected by changes in resting‐state electroencephalographic (rsEEG) rhythms recorded during eyes closed, psychophysiological relaxation, and mind wandering.^27^ In cognitively healthy older adults, rsEEG alpha rhythms (8–12 Hz) predominate in the posterior scalp regions, whereas rsEEG delta (< 4 Hz) and theta (4–7 Hz) rhythms are typically widespread and low in amplitude.^28^ In contrast, ADMCI and ADD patients exhibit a “slowing” of that EEG activity with increased widespread delta (< 4 Hz) and theta (4–7 Hz) rhythms associated with decreased alpha (8–13 Hz) rhythms in posterior cortical regions.^29^ This “slowing” has been associated with cerebrospinal fluid (CSF) tau levels,^28^, ^29^, ^30^ *apolipoprotein E4 (APOE4)* genotype,^31^, ^32^, ^33^ and low cholinergic activity,^34^ and was observed in older individuals with subjective memory complaint (SMC) associated with the risk of progression to MCI,^35^, ^36^ hippocampal atrophy, and brain hypometabolism.^37^

Previous INSIGHT project studies unveiled the educational attainment effects on rsEEG rhythms in SMC individuals positive (SMCpos) and negative (SMCneg) to ^18^F‐florbetapir PET biomarkers probing brain amyloidosis load as an index of early Alzheimer's pathology.^38^, ^39^ The SMCneg Edu+ (no preclinical AD) participants showed the greatest posterior rsEEG alpha rhythms, possibly reflecting neuroprotective mechanisms,^38^ and an association between these rhythms and the thalamic functional connectivity with cortical visual networks, as measured by resting‐state functional MRI (rs‐fMRI).^39^ Conversely, the SMCpos Edu+ (preclinical AD) participants showed the lowest posterior rsEEG alpha rhythms, possibly reflecting compensatory mechanisms^40^ and disruption of that functional association.^39^ Similar results were obtained in ADMCI patients from the PDWAVES project^40^.

The present INSIGHT project study tested whether brain structure from MRI and the above compensatory educational attainment effects on posterior rsEEG rhythms are stable in the SMCpos Edu+ particpants during the typical duration of a clinical prevention or intervention study, that is, 12 and 24 months. This issue is relevant to better understanding the possible disease progression at earlier stages of Alzheimer's pathology in SMC people. It has ethical implications for communications with them and their families.

**RESEARCH IN CONTEXT**

1. **Systematic review**: Previous studies have explored cognitive reserve (CR) role in mitigating Alzheimer's disease (AD) pathology and in delaying cognitive decline. No longitudinal research has investigated how educational attainment—a key CR component—interacts with preclinical amyloid‐β accumulation and resting‐state electroencephalographic (rsEEG) rhythms, reflecting vigilance dysfunction in preclinical AD.
2. **Interpretation**: We demonstrated that the compensatory effects of educational attainment on posterior rsEEG alpha rhythms and brain structure were stable over 2 years in older adults with subjective memory complaints (SMC) and amyloid‐β pathology. These stable alterations of rsEEG alpha rhythms suggest that vigilance dysfunctions (like diurnal drowsiness, mental fatigue) may precede measurable cognitive decline associated with AD pathophysiology.
3. **Future directions**: Larger, more diverse cohorts and longer follow‐ups are needed to clarify how lifestyle and demographics affect CR‐related neuroprotective and compensatory mechanisms. EEG could enrich Precision Medicine approaches for detecting and addressing early vigilance impairments in individuals at risk for AD.

## 2 METHODS

The present retrospective and exploratory study is based on pre‐existing data from the INSIGHT Consortium.^41^, ^42^ The procedures concerning the participants’ enrollment, PET to measure brain amyloidosis, MRI, and rsEEG recordings acquisition and preprocessing are detailed in.^38^, ^39^ We report a recap of the main procedures in the following sections.

### 2.1 Participants

Participants were recruited at the Pitié‐Salpêtrière University Hospital in Paris, France, in the framework of the INSIGHT‐preAD study to investigate the earliest preclinical stages of AD and its development, including influencing factors and markers of disease progression.^41^, ^42^ The INSIGHT‐preAD study currently includes baseline data in 318 cognitively intact individuals, between 70 and 85 years old, with SMC status confirmed by an affirmative answer to both of the following questions: (1) “Are you complaining about your memory?” and (2) “Is it a regular complaint which lasts more than 6 months”? Indeed, cognition was unimpaired as revealed by a Mini‐Mental State Examination (MMSE;^43^) score ≥ 27, Clinical Dementia Rating^44^ score equal to 0, and no evidence of episodic memory deficit measured by the total recall at the Free and Cued Selective Reminding Test (mean 46.1 ± 2.0;^45^).

At the baseline recording session (M0), demographic, cognitive, functional, nutritional, biological, genetic, genomic, imaging, and electrophysiological data were collected (see^38^, ^39^, ^41^, ^42^ for details). The same measurements were repeated after 12 months (cognitive and electrophysiological data; M12) and after 24 months (cognitive, electrophysiological data, and imaging data; M24).

All experiments were performed with each participant or caregiver's informed and overt consent, per the Code of Ethics of the World Medical Association (Declaration of Helsinki) and the standards established by the local Institutional Review Board at the participating center (Ethical approval number: 2013‐Fev‐13150). All participants or their representatives gave written informed consent for the use of their clinical data for research purposes. Participants were recruited without discrimination based on gender, ethnicity, socioeconomic status, or other personal characteristics, ensuring a diverse and representative sample.

### 2.2 Aβ PET (^18^F‐florbetapir PET) data acquisition and processing for participants' grouping

Participants with SMC were stratified into two groups of amyloid‐positive (SMCpos) and ‐negative (SMCneg), using the standard diagnostic markers of Alzheimer's neuropathology based on cortical‐to‐cerebellum standardized uptake value ratio (SUVR) in that PET imaging.

PET scans were acquired 50 min after injection of 370 MBq (10 mCi) ^18^F‐florbetapir. Reconstructed PET images were analyzed with a pipeline developed by the Centre d'Acquisition et Traitement des Images (CATI) (http://cati‐neuroimaging.com). Structural MRI images were co‐registered to ^18^F‐florbetapir Aβ PET images using SPM8 (https://www.fil.ion.ucl.ac.uk/spm/software/spm8/) with visual inspection to detect any co‐registration errors. Inverse deformation fields and matrix transformation from MRI data processing were used to derive composite cortical regions of interest (ROIs; left and right precuneus, posterior and anterior cingulate, parietal, temporal, and orbitofrontal cortex, according to^46^) and a reference region (in the pons and whole cerebellum) were placed in the individual native PET space. The region‐based voxel‐wise correction symmetric geometric transfer matrix (RBV‐sGTM) method was used to correct for the partial volume effect.^47^ Parametric PET images were created for everyone by dividing each voxel by the mean activity extracted from the reference region.

For Aβ ^18^F‐florbetapir PET images, we calculated  SUVRs by averaging the mean activity of cortical regions of interest: left and right precuneus, cingulum posterior, cingulum anterior, and parietal, temporal, and orbitofrontal cortices.^41^ The reference region was a combination of the whole cerebellum and pons regions. The SUVR threshold to determine abnormal uptake was extracted by linear correlation between the CATI's method and the one used by Besson and colleagues for 53 PET scans (26 elderly healthy controls, 11 patients with mild cognitive impairment, and 16 patients with clinical probable AD) obtained from the Multimodal Imaging of Early‐Stage Alzheimer's Disease cohort (see^41^ appendix for the reference studies). The threshold set for positive versus negative Aβ deposition was 0.7918 (see^41^ appendix for details), resulting in the following groups: SMCneg, *N* = 230, SMCpos, *N* = 88.^48^ Neither the participants nor the investigators were aware of the participants' Aβ status.

### 2.3 Stratification of the SMC seniors based on the educational attainment

As a proxy of the cognitive reserve, we used the education score adopted in the formal INSIGHT‐preAD project protocol.^41^ In this score, the level of education ranged from 1 to 8; 1 means the attendance of the only infant school (8 years of education), and 8 means the attendance of a higher education level in the population (i.e., bachelor, master's degree, or doctorate; at least 16 years of education). Based on this score, all the present SMC participants were stratified according to the median value in the two sub‐groups. SMC participants with a low‐to‐moderate education level, from 1 to 6 of the education score, were denoted as SMC Edu‐. In contrast, SMC participants with education scores ranging from 7 to 8 were marked as SMC Edu+^38^, ^39^.

### 2.4 MRI acquisition and preprocessing

For the cortical signature of the SMC participants, MRI acquisitions of the brain were conducted using a 3 Tesla scanner with parallel imaging capabilities (Siemens Magnetom Verio, Siemens Medical Solutions, Erlangen, Germany). The scanner used a quadrature detection head coil with 12 channels (transmit–receive circularly polarized [CP] head coil). For the anatomical study, 3D TurboFLASH sequences were performed (orientation sagittal; repetition time [TR] = 2300 ms; echo time [TE] = 2.98 ms; inversion time = 900 ms; flip angle = 9°; 176 slices; slice thickness = 1 mm; field of view = 256*240 mm; matrix = 256*240; bandwidth = 240 Hz/Px). Cavedo et al.^49^ reported the technical details concerning the preprocessing for the cortical signature of prodromal AD and the automated calculation of hippocampus (HP) and basal forebrain (BF) volumes.

### 2.5 rsEEG recordings

EEG data were recorded while the participants sat comfortably and relaxed with eyes closed in a standard resting‐state condition. At least 120 seconds of rsEEG data (e.g., two periods of eyes‐closed condition lasting 30 s each, intermingled with two periods of eyes‐open condition) were acquired using a high‐density 256‐channel EGI system (Electrical Geodesics Inc., USA) with a sampling rate of 250 Hz and anti‐aliasing bandpass analogic filtering. In this EGI system, the electrodes used are sponge‐based to have a quick application time (10–20 min), which is ideal for seniors. Among the 256 electrodes, 224 electrodes cover the whole scalp. In contrast, the remaining ones are placed on the front, the top of the neck, and the face, allowing the measurement of electro‐oculographic (EOG) and muscular electromyographic (EMG) activity. The impedances of all scalp electrodes were kept below 50 KΩ. The reference electrode was placed at the Cz site. Cephalic ground was used.

### 2.6 Spectral and quality analysis of the rsEEG data

In line with a previous reference study using the same rsEEG database,^38^, ^39^ we used the rsEEG activity recorded from 68 out of 256 scalp electrodes, namely those showing the best quality of the collected data. Figure S1 illustrates the spatial distribution on the scalp of these 68 electrodes.

**FIGURE 1:**
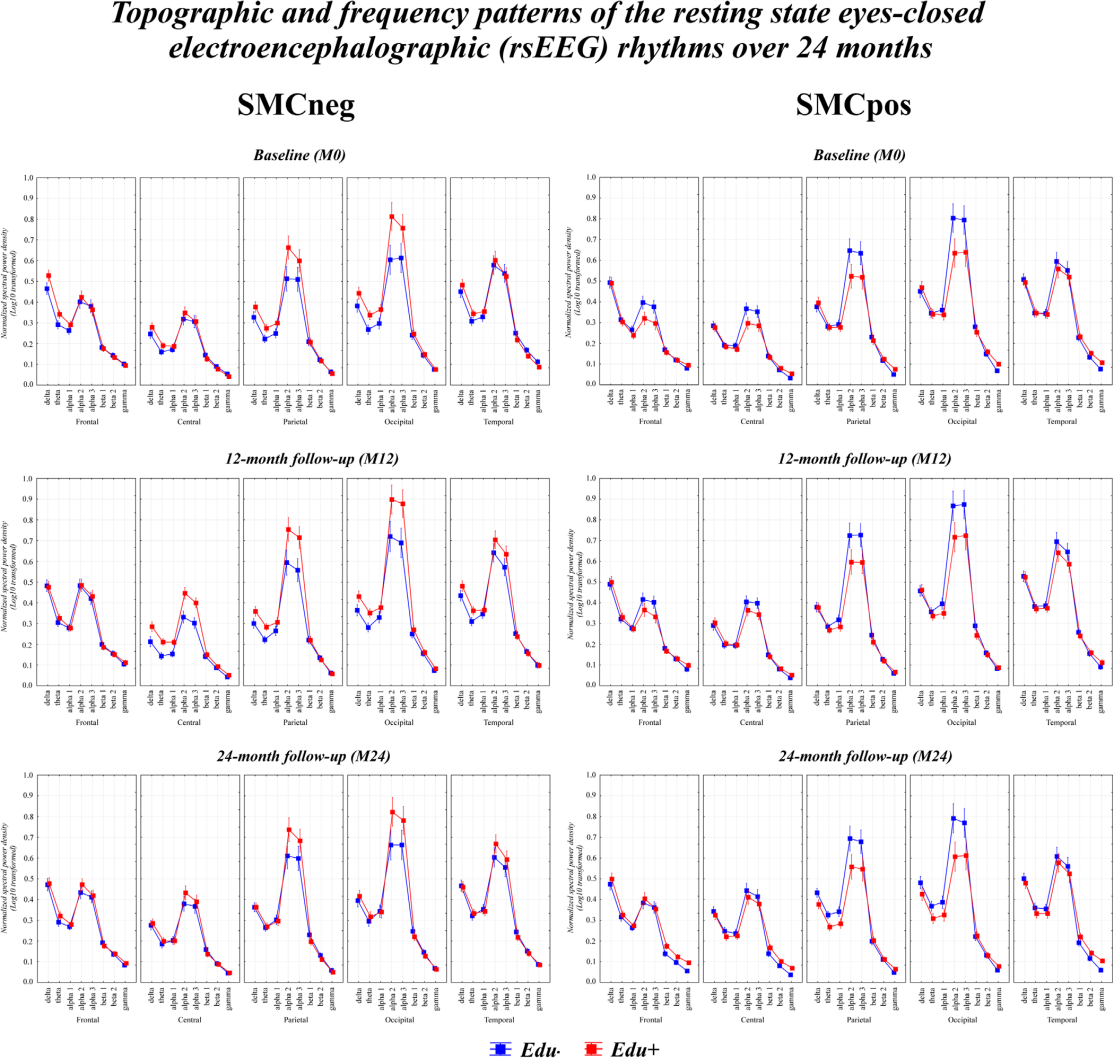
Topographic and frequency patterns of the resting‐state eyes‐closed electroencephalographic (rsEEG) rhythms over 24 months. Mean values (± standard error mean, SE) of power density in normalized rsEEG rhythms in seniors with subjective memory complaint (SMC) found to be amyloid negative (SMCneg; 1a) and positive (SMCpos; 1b) to the marker of Alzheimer's neuropathology derived from ^18^F‐florbetapir positron emission tomography (amyloid‐β PET). These data are ordered for (1) low or higher educational attainment level (Edu‐ and Edu+), (2) five regions of interest (ROIs) of the scalp (frontal, central, parietal, occipital, and temporal), (3) eight frequency bands of rsEEG rhythms (delta, theta, alpha 1, alpha 2, alpha 3, beta 1, beta 2, and gamma), and (4) three timepoints, namely the baseline (M0), 12‐month follow‐up (M12), and 24‐month follow‐up (M24). No statistically significant effect was produced by the analysis of variance (ANOVA) design (*p* > 0.05) using the factors Time, Group, Education, Band, and ROI). Across the time points, in all sub‐groups, the posterior (i.e., parietal, temporal, and occipital) regional rsEEG power density spectra were characterized by well‐shaped rsEEG alpha peaks and other benchmark features of rsEEG recordings of sufficient quality such as (1) Visible TFs and IAFps; (2) Drop of rsEEG power density values as a function of the increase of rsEEG frequencies after alpha power density peaks; (3) No significant power density offsets at gamma frequencies in some ROIs; and (4) No multiple rsEEG power density peaks in the spectra. IAFp, individual alpha frequency peak; SMC, subjective memory complaint negative (SMCneg) or positive (SMCpos) to amyloid load; TF, transition frequency.

The preliminary rsEEG data analysis aimed at minimizing the inter‐individual variance of rsEEG rhythms to diminish (1) confounding effects of border electrodes with high impedance in the electrode montage; (2) rsEEG data with residual artifacts provoked by the head or eye muscle activities; and (3) individual variability in the rsEEG alpha frequency power peak (i.e., using individual alpha frequency peak [IAFp] as a benchmark;^50^). After this “pruning” procedure, 42 SMCneg and 42 SMCpos seniors with MMSE scores ≥ 28 were associated with accepted rsEEG datasets for all the time points (M0, M12, and M24). They also had available structural MRI data.

The rsEEG frequency bands of interest were individually determined based on the following frequency landmarks: the transition frequency (TF) and the IAFp.^50^ The TF marks the transition frequency between the rsEEG theta and alpha bands in the power density spectra. Specifically, the TF was defined as the frequency showing minimum rsEEG power density between 3 and 8 Hz (i.e., between the delta and the alpha power peak). The IAFp was defined as the frequency showing maximum power density peak between 6 and 14 Hz. The analysis of the TF and IAFp was performed on rsEEG power density averaged across Fz, Cz, Pz, O1, and O2 scalp electrodes. These electrodes typically show EEG delta, theta, and alpha rhythms with low biological noise (e.g., the scalp midline is relatively far from temporal and frontal muscles) and optimal electrical contacts using a standard EEG helmet.^38^, ^39^

In detail, the individual frequency bands from delta to alpha were determined as follows, from rsEEG power density spectra computed with a frequency resolution of 0.5 Hz:

1. Elta from TF ‐4 Hz to TF ‐2 Hz.
2. Theta from TF ‐2 Hz to TF.
3. Alpha 1 from TF to the midpoint of the TF‐IAFp range.
4. Alpha 2 from the middle of the TF‐IAFp range to IAFp.
5. Alpha 3 from IAFp to IAFp + 2 Hz.

An example of the rsEEG power density with the indication of the TF, IAFp, and the individual frequency band I illustrated in Figure S2.

In the main analysis, we focused on the individual rsEEG delta, theta, and alpha frequency bands because a mean slowing in the peak frequency of the alpha power density may characterize a clinical group without any substantial change in the magnitude of the power density. In that case, using fixed frequency bands would result in a statistical effect erroneously showing rsEEG alpha power density values lower in the clinical than in the control group.

No statistically significant effect, including the Time or the two‐way Group (SMCneg, SMCpos) × Edu (Edu‐, Edu+) interaction (*p* > 0.05), was observed for the TF and IAFp. The longitudinal TF and IAFp values for each sub‐group are reported in the Supplementary Material Results.

The statistical analysis also considered fixed beta 1 (13–20 Hz), beta 2 (20–30 Hz), and gamma (30–40 Hz) bands.

Five regions of interest (ROIs) were used to consider the frontal, central, parietal, occipital, and temporal electrodes of the 10‐10 electrode montage system. Specifically, these ROIs included the electrodes reported in Table S1. Evaluating the whole‐scalp topographic rsEEG patterns allowed us to evaluate the overall final quality and compare the results with previous studies.

### 2.7 Statistical analysis of rsEEG power density, neuroimaging, and neuropsychological variables

To test the study hypotheses, the commercial tools STATISTICA 10 (StatSoft Inc., www.statsoft.com) and RStudio (https://posit.co/downloads/) were used in three statistical sessions. Due to the factorial design of the present study, we used the linear general model and ANOVAs for the comparison of the rsEEG power density, neuroimaging (Aβ PET and sMRI), and the neuropsychological variables between the SMC sub‐groups. The Shapiro–Wilk and Kolmogorov–Smirnov tests were used to determine if the variable distributions of a given ANOVA model approximated Gaussian distributions (null hypothesis of non‐Gaussian distributions tested at *p* < 0.05). The rsEEG power density distributions were processed by the Log10 transformation, approximating a Gaussian distribution. The MRI markers and neuropsychological scores distribution already satisfied the assumption of normality (*p* > 0.05). The rsEEG, MRI, and neuropsychological variables distribution were also visually checked with the QQ plots. The degrees of freedom were corrected by the Greenhouse–Geisser procedure when appropriate. The Duncan test was used for post‐hoc comparisons using a statistical threshold of *p* < 0.05 corrected for the planned contrasts. The planned contrasts focused on the comparison of the rsEEG, neuroimaging, and neuropsychological measures (1) between the SMCneg and SMCpos sub‐groups and (ii) between the Edu‐ and Edu+ sub‐groups (*p* < 0.05 Bonferroni corrected).

The first statistical session was performed to evaluate the hypothesis that the posterior rsEEG power density at the individual alpha 2 and alpha 3 bands might differ (1) between the different time points (M0, M12, M24), (2) between the SMCneg and SMCpos sub‐groups, and (3) between the Edu‐ and Edu+ sub‐groups of the SMCneg and SMCpos seniors (*p* < 0.05 corrected). To this aim, an ANOVA was used with the regional rsEEG power density as the dependent variable. The ANOVA factors were Time (M0, M12, M24), Group (SMCneg and SMCpos), Education (Edu‐ and Edu+), Band (delta, theta, alpha 1, alpha 2, alpha 3, beta 1, beta 2, and gamma), and ROI (frontal, central, parietal, occipital, and temporal).

The second statistical session was performed to evaluate the hypothesis that the brain amyloidosis and volumetric brain integrity, as respectively measured by Aβ PET and structural MRI data, might differ (1) between the different time points (M0, M24), (2) between the SMCneg and SMCpos sub‐groups, and (3) between the Edu‐ and Edu+ sub‐groups of the SMCneg and SMCpos seniors (*p* < 0.05 corrected). To achieve this aim, several ANOVAs were performed, one for each Aβ PET and MRI measure as dependent variables. The ANOVA factors were Time (M0, M24), Group (SMCneg and SMCpos), and Education (Edu‐ and Edu+).

The third statistical session was performed to evaluate the hypothesis that the cognitive‐functional abilities as measured by the neuropsychological tests might differ (1) between the different time points (M0, M12, M24), (2) between the SMCneg and SMCpos sub‐groups, and (3) between the Edu‐ and Edu+ sub‐groups of the SMCneg and SMCpos seniors (*p* < 0.05 corrected). To this aim, several ANOVAs were performed, one for neuropsychological test scores as the dependent variable. The following neuropsychological tests were considered: MMSE, DMS 48^51^ learning, DMS 48 immediate recognition (time), DMS 48 immediate recognition score, DMS 48 1‐h recognition (time), DMS 48 1‐h recognition score, Trial Making test (TMT;^52^) B‐A, Copy of the Rey figure^53^ immediate (time), Copy of the Rey figure after 3 min (time), and Copy of the Rey figure after 30 min (time). The ANOVA factors were Time (M0, M12, M24), Group (SMCneg and SMCpos), and Education (Edu‐ and Edu+).

## 3 RESULTS

### 3.1 Characterization of demographic, neuropsychological, and genetic markers in the SMCneg and SMCpos groups (based on amyloid PET biomarkers) and the effect of education attainment

Table 1 reports the relevant demographic, neuropsychological (MMSE score), and genetic (*APOE* genotype) markers in the SMCneg Edu‐, SMCneg Edu+, SMCpos Edu‐, and SMCpos Edu+ sub‐groups. It also reports the results of the statistical analyses (*p* < 0.05) computed to evaluate the presence or absence of statistically significant differences between the Edu‐ and Edu+ sub‐groups for both SMCneg and SMCpos seniors as age (*t*‐test), sex (Fisher test), education (*t*‐test), MMSE score (Mann–Whitney *U* test), *APOE* genotype (chi‐squared test), and *APOE* e4 carriers/non‐carriers. Based on the stratification criterion, as expected, a statistically significant difference in education was found between the Edu‐ and Edu+ sub‐groups for both SMCneg and SMCpos seniors considered separately (*p* < 0.00001). On the contrary, no statistically significant difference was found for the age, sex, MMSE score, *APOE* genotype, and *APOE* e4 carriers/non‐carriers between the Edu‐ and Edu+ sub‐groups for both SMCneg and SMCpos seniors considered separately (*p* > 0.05).

**TABLE 1:** Mean values (± standard error mean, SE) of the demographic, neuropsychological, and genetic data (presence or absence of at least one ε4 allele), together with the results of their statistical comparisons (*p* < 0.05) in the groups of seniors with subjective memory complaint (SMC) found to be amyloid‐β negative (SMCneg) and positive (SMCpos) to the marker of preclinical Alzheimer's neuropathology derived from ^18^F‐florbetapir positron emission tomography (amyloid‐β PET).

Parameter	SMCneg Edu‐	SMCneg Edu+	SMCpos Edu‐	SMCpos Edu+	Statistical comparison (Edu‐ ≠ Edu+)
*N*	20	22	20	22	‐
Age	75.0 ± 0.7	75.7 ± 0.6	76.6 ± 0.6	75.3 ± 0.8	*t*‐test: n.s
Sex (M/F)	4/16 (20%)	11/11 (50%)	5/15 (55%)	10/12 (45%)	Fisher test: n.s.
Education	4.5 ± 0.3	7.9 ± 0.07	4.1 ± 0.3	7.7 ± 0.3	***t*‐test**: **Edu+ > Edu‐ =** *p* **< 0.001**
MMSE	28.6 ± 0.2	29.0 ± 0.2	28.7 ± 0.2	29.0 ± 0.2	Mann–Whiney *U* test: n.s
***APOE* ε4** **(Y/N)**	5/15 (20%)	5/17 (27%)	8/12 (40%)	6/16 (27%)	Fisher test: n.s

Abbreviations: *APOE*, apolipoprotein E; M/F, males/females; Edu+ and Edu‐, higher and lower, respectively, education level attained; MMSE, Mini‐Mental State Examination; n.s., not significant (*p* > 0.05); SMC, subjective memory complaint negative (SMCneg) or positive (SMCpos) to amyloid load; Y/N, yes/no.

### 3.2 Longitudinal comparisons in the regional rsEEG alpha power density between the SMCneg and SMCpos sub‐groups and the effect of educational attainment

No significant Time × Group × Education × Band × ROI interaction was found (*p* > 0.05). Across time points, all sub‐groups displayed typical rsEEG alpha patterns in posterior regions and other benchmark features of sufficient‐quality rsEEG recordings (see Figure 1). These results highlighted that no differences in the topographic and frequency distribution of the rsEEG power density in the SMCneg and SMCpos sub‐groups may be appreciated across the different time points.

**FIGURE 2:**
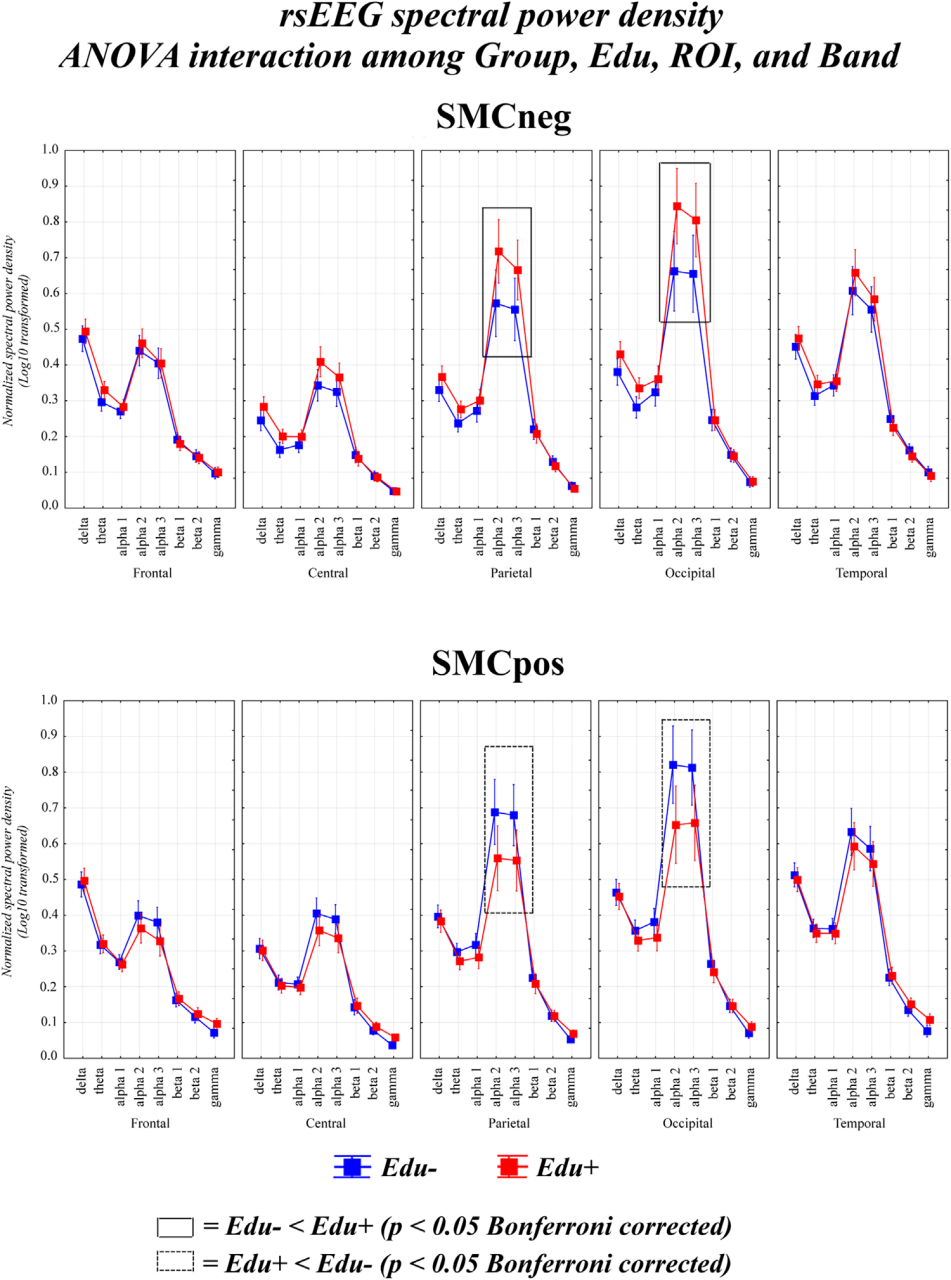
Effect of education on the rsEEG rhythms in the SMCneg and SMCpos seniors. Mean values (± standard error mean, SE) of normalized rsEEG power density for (1) two groups (SMCneg and SMCpos), (2) two education levels (higher [Edu+] and lower [Edu‐]), (3) five ROIs (frontal, central, parietal, occipital, and temporal), and (4) eight frequency bands (delta, theta, alpha 1, alpha 2, alpha 3, beta 1, beta 2, and gamma). The ANOVA showed a statistically significant 3‐way Group × Education × Band × ROI interaction effect (F (28, 2240) = 4.87, *p* < 0.00001). The rectangles indicate the scalp regions and frequency bands in which the rsEEG power density presented a statistically significant pattern: Edu+ ≠ Edu‐ in the SMCneg and SMCpos senior groups (Duncan post‐hoc test, *p* < 0.05 corrected for multiple comparisons = *p* < 0.0025; see “Methods” for the criterion of such correction). rsEEG, resting‐state electroencephalographic; ROI, region of interest; SE, standard error; SMC, subjective memory complaint negative (SMCneg) or positive (SMCpos) to amyloid load.

A significant four‐way Group × Education × Band × ROI interaction emerged (F (28, 2240) = 4.87, *p* < 0.00001; see Figure 2). The Duncan planned post‐hoc comparisons showed that in the SMCneg group, higher education was associated with increased parietal and occipital rsEEG alpha 2 and alpha 3 power density (*p* < 0.0025, equivalent to *p* < 0.05 corrected). In contrast, in the SMCpos group, higher education was linked to lower rsEEG power density in the same regions and bands. These differences were not due to outliers (Grubbs’ test at *p* < 0.001) or variability in the number of usable rsEEG epochs between Edu‐ and Edu+ sub‐groups (*t*‐test; *p* > 0.05).

### 3.3 Longitudinal comparisons of the brain amyloidosis and integrity between the SMCneg and SMCpos sub‐groups and the effect of educational attainment

**FIGURE 3:**
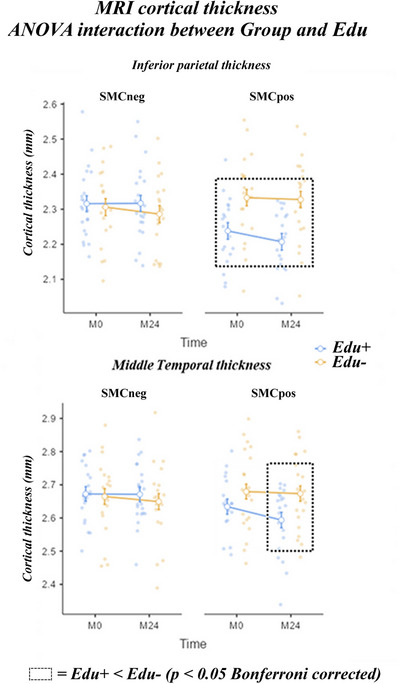
Effect of education on the MRI variables in the SMCneg and SMCpos seniors. Mean values (± standard error mean, SE) of the cortical inferior parietal (upper) and middle temporal (lower) thickness for: (1) two groups (SMCneg and SMCpos), (2) two Education levels (higher [Edu+] and lower [Edu‐]), and (4) two timepoints, namely the baseline (M0) and 24‐month follow‐up (M24). No statistically significant effect was produced by the ANOVA design (*p* > 0.05) using the factors Time, Group, and Edu. The ANOVA showed a statistically significant 2‐way Group × Education interaction (F (1, 78) = 7.74, *p* < 0.005 for the inferior parietal thickness; F (1, 78) = 2.96, *p* < 0.05 for the middle temporal thickness). Only for the SMCpos seniors, a statistically significant difference Edu+ ≠ Edu‐ was observed both at the M0 and M24 timepoints for the inferior parietal thickness and only at M24 for the middle temporal thickness (Duncan post‐hoc test, *p* < 0.05 corrected for multiple comparisons = *p* < 0.0125; see “Methods” for the criterion of such correction). ANOVA, analysis of variance; MRI, magnetic resonance imaging; SE, standard error; SMC, subjective memory complaint negative (SMCneg) or positive (SMCpos) to amyloid load.

No significant three‐way Time × Group × Education interaction was observed for Aβ PET and MRI variables (*p* > 0.05). A statistically significant two‐way Group × Education interaction was found for the inferior parietal (F (1, 78) = 7.74, *p* < 0.005) and middle temporal (F (1, 78) = 2.96, *p* < 0.05) cortical thickness from MRIs (see Figure 3).

The Duncan planned post‐hoc comparisons (*p* < 0.0125, equivalent to *p* < 0.05 corrected) showed no differences between the SMCneg sub‐groups. Compared to the SMCpos Edu‐sub‐group, the SMCpos Edu+ sub‐group exhibited lower inferior parietal thickness (*p* < 0.001) both at M0 and M24 and lower middle temporal thickness at M24 (*p* < 0.001). The above effects of the education level were not due to the presence of outliers (statistical threshold of Grubbs’ test at *p* < 0.001).

Non‐significant models are reported in Figure S3. The main Group effect (*p* < 0.05) suggested greater brain amyloidosis and neurodegeneration in the SMCpos compared to SMCneg participants at both time points.

### 3.4 Longitudinal comparisons in the cognitive‐functional abilities between the SMCneg and SMCpos sub‐groups and the effect of educational attainment

No significant three‐way Time × Group × Education interaction was found for the neuropsychological variables (*p* > 0.05). The results of non‐significant models are in Figure S4. Mostly, the Group effect was statistically significant (*p* < 0.05), suggesting that the SMCpos participants were characterized by higher visual recognition impairment as compared to the SMCneg seniors at both M0, M12, and M24, stable over time.

### 3.5 Control analysis on the association between rsEEG alpha rhythms, Aβ PET‐MRI markers, and neuropsychological tests over 24 months

For cross‐validation purposes, we tested the associations among the neuroimaging data, the posterior rsEEG alpha rhythms, and the cognitive‐functional abilities measured by neuropsychological tests. To this purpose, we performed a general linear regression analysis at M0 and M24 (neuroimaging‐rsEEG variables) and M0, M12, and M24 (rsEEG‐neuropsychological variables).

The first (control) cross‐validation analyses included several linear regression models (one model for each rsEEG and the corresponding neuroimaging variable at M0 and M24) having the following features.

1. Dependent rsEEG variables: parietal and occipital rsEEG alpha 2 and alpha 3 power density (one model for each variable).
2. Predictors: Group (SMCneg, SMCpos), Education (Edu+, Edu‐), Neuroimaging variable (brain amyloidosis load measured by PET and MRI‐based normalized gray matter volume, normalized white matter volume, normalized CSF volume, BF volume, HP volume, entorhinal cortex volume, entorhinal cortex thickness, fusiform thickness, inferior parietal thickness, inferior temporal thickness, lateral occipital thickness, middle temporal thickness, para‐hippocampal thickness, paracentral thickness, pericalcarine thickness, postcentral thickness, posterior cingulate thickness, precentral thickness, precuneus thickness, superior frontal thickness, superior parietal thickness, superior temporal thickness, insula thickness, white matter hyperintensities; one model for each variable), and two‐ and three‐way interactions among Group, Education, and MRI variables.

No statistically significant three‐way Group × Education × neuroimaging (amyloid PET or MRI) variable interaction was observed, both at M0 and M24 (*p* > 0.05). There were only statistically significant main and two‐way Group × BF volume interactions, both at M0 and M24, in the association with the parietal and occipital rsEEG alpha 2 and alpha 3 power density (*p* < 0.02; see Tables S2 and S3 for details). The post‐hoc analysis revealed that, only in the SMCneg participants, there was a statistically significant (*p* < 0.05, false discovery rate [FDR] corrected) positive association between the BF volume and the parietal and occipital rsEEG alpha 2 power density at M0 (Figure S5). The same patterns were observed at M24 (Figure S6) for the parietal and occipital rsEEG alpha 2 and alpha3 power density.

The second (control) cross‐validation rsEEG analysis included several linear regression models (one model for each rsEEG and the corresponding neuropsychological scores at M0, M12, and M24) having the following features.

1. Dependent neuropsychological score: MMSE, DMS 48 learning, DMS 48 immediate recognition (time), DMS 48 immediate recognition score, DMS 48 1‐h recognition (time), DMS 48 1‐h recognition score, Trial Making test (TMT) B‐A, Copy of the Rey figure immediate (time), Copy of the Rey figure after 3 min (time), and Copy of the Rey figure after 30 min (time; one model for each variable).
2. Predictors: Group (SMCneg, SMCpos), Education (Edu+, Edu‐), rsEEG variables (parietal and occipital alpha 2 and alpha 3 power density; one model for each variable), and two‐ and three‐way Group × Education × rsEEG variables interactions.

The results showed statistically significant three‐way Group × Education × rsEEG variables (parietal and occipital rsEEG alpha 2 and alpha 3 power density) interaction in the association with the DMS 48 1‐h recognition (time) and the Copy of the Rey figure immediate (time) in the SMC participants, both at M0 (Table S4) and M12 (Table S5). No statistically significant three‐way Group × Education × rsEEG variables interactions were observed at M24 (*p* > 0.05). These results indicate that in the SMCpos participants with low educational attainment, higher posterior rsEEG alpha rhythms were predictive of better visual memory performance, thus suggesting that CR may compensate for the initial abnormalities in the posterior rsEEG alpha rhythms in SMCpos people. The post‐hoc analysis revealed that, only in the SMCpos Edu‐ sub‐group, there was a statistically significant (*p* < 0.05, FDR corrected) negative association between the parietal and occipital rsEEG alpha 2 and alpha 3 power density and the Copy of the Rey figure immediate (time) at M0 (Figure S7) and M12 (Figure S8). No statistically significant results were observed for M24.

### 3.6 Control analysis on the brain tauopathy in the SMCneg and SMCpos seniors

In line with the hypothesis that Alzheimer's brain amyloidosis load is associated with and may drive the spreading of greater brain tau pathology, we performed a control analysis. Specifically, we evaluated the Aβ1‐42, phospho‐tau, and total‐tau measures in the CSF in relation to the amyloidosis revealed by Aβ PET. Unfortunately, these data were available only for a minority of the SMC participants enrolled in the present study (SMCneg, *N* = 7, SMCpos, *N* = 11). Several *t*‐tests were performed (SMCneg ≠ SMCpos; *p* < 0.05), having the CSF measure of Aβ1‐42, total‐tau, phospho‐tau, ratio between the total‐tau/Aβ1‐42, and ratio between the phospho‐tau/Aβ1‐42 as dependent variables.

Results showed that, as expected, the SMCpos seniors were characterized by lower Aβ1‐42, higher total‐ and phospho‐tau, as well as higher total‐tau/Aβ1‐42 and phospho‐tau/Aβ1‐42 values in the CSF as compared to the SMCneg seniors (*p* < 0.05; see Figure S9).

### 3.7 Control analysis using educational attainment as a continuous variable

We performed several regression models to confirm the main results of the rsEEG and MRI variables using continuous educational levels.

The first regression model used the posterior rsEEG alpha power density at the different timepoints (M0, M12, and M24) as the dependent variable and the educational attainment (as a continuous variable) and Group (SMCneg, SMCpos) as predictors. The second regression model used the neuroimaging measures at the different timepoints (M0 and M24) as the dependent variable and the educational attainment (as a continuous variable) and Group (SMCneg, SMCpos) as predictors. The results are reported in a control section of the Supplementary Material Results. There was a statistically significant two‐way Group × Education interaction (*p* < 0.05) in the association with the parietal and occipital rsEEG alpha 2 and alpha 3 power density at all the timepoints (M0, M12, M24), in line with the main data analysis. However, the post‐hoc analysis revealed no statistically significant Group differences (*p* > 0.05, FDR corrected), possibly due to the impact of educational attainment levels (e.g., high school degree) on subsequent working activity rather than the years of educational attainment per se. Concerning the MRI variables, only for the fusiform thickness at M0 and for the inferior parietal thickness at M24, there was a statistically significant two‐way Group × Education interaction (*p* < 0.05). The details of the results are reported in Table S6 and Figures S10 and S11.

### 3.8 Control analysis on the adapted version of the short Cognitive Reserve Index questionnaire

Educational attainment partially represents CR, as it does not consider work duration and complexity of lifelong intellectual activities, general cultural experience, and creative/leisure pursuits. In this framework, occupational complexity has raised attention in considering the impact of CR on neurodegenerative diseases. It may confer additional resilience to the adverse effects of AD neuropathology on cognition (e.g., see Supplementary Material Reference^3^, ^4^). Unfortunately, in the present study, information on the whole duration of the working activities over the lifespan was not available. Furthermore, the qualifications of SMC participants’ job complexity and specific details of the leisure activities (e.g., attending social events, theaters, conferences, etc.) were not available either.

To partially expand the concept of CR, we performed two control analyses using the short Cognitive Reserve Index questionnaire (CRIq; Supplementary Material Reference^5^) as a conceptual reference to enrich the educational attainment as a proxy of CR. Roughly, we estimated the duration of the working activities by subtracting the age of completion of educational studies (not lower than 18 years old) from the age at cessation of working activities. For the job qualification, we used the standard employee level for all the SMC cohort. Based on these criteria, we roughly estimated the CRI. We stratified the SMCneg and SMCpos participants according to the median value into low (CRI−) and high (CRI+) CRIq (see Table S7 for the variables used and SM8 for the clinical‐demographic characteristics of each sub‐group). The results are reported in detail in the Supplementary Material Results and are summarized in the following.

The statistical analysis revealed a complex interaction between group classification (based on amyloid status), CRI, rsEEG frequency bands, and brain regions (Figure S12). In the SMCneg sub‐group, higher over lower CRI was significantly associated with greater rsEEG alpha rhythms (alpha 2 and alpha 3) in parietal and occipital regions (*p* < 0. 0001), potentially reflecting a neuroprotective factor. Conversely, in the SMCpos participants, higher over lower CRI was linked to reduced rsEEG alpha rhythms in the same regions (*p* < 0. 0005), suggesting a different or possibly compensatory mechanism at play. Along the same line, a significant interaction between Group and CRI was observed in the inferior parietal and middle temporal regions measured with the structural MRIs. This effect was especially evident in the SMCpos sub‐group, where higher over lower CRI was associated with thinner cortex in the inferior parietal region (*p* < 0.0125; Figure S13). Finally, no significant interactions were observed between amyloid group status and CRI on cognitive status in the SMC participants.

Overall, the control analyses' findings support the idea that CR interacts with brain function (rsEEG measures) and structure (MRI measures), and that the kind of interaction is quite similar using proxies only based on educational attainment or with educational attainment enriched with other rough sources of information about SMC participants’ working activities. This would be due to the intrinsic influence of educational attainment on the features of working activities along the lifespan.

### 3.9 Control analysis on the correlation between rsEEG theta, alpha, and gamma rhythms and amyloid quantified by SUVR PET

The main results showed an interaction of the effects of educational attainment and brain amyloid load on rsEEG alpha rhythms in the SMC participants. However, EEG theta and gamma rhythms are considered important in relation to Alzheimer's pathology in human and animal models (see Supplementary Material References^6^, ^7^). For exploratory purposes, we used the ratio between the rsEEG theta and alpha 2 power density and the ratio between the rsEEG theta and alpha 3 power density as dependent variables in two ANOVA designs having the Time (M0, M12, M24), Group (SMCneg, SMCpos), Education (Edu‐, Edu+), and ROI (frontal, central, parietal, occipital, temporal) as factors. We then performed a control analysis to test the association between baseline rsEEG theta, alpha, theta/alpha 2, theta/alpha 3, and gamma and the brain amyloid load as measured by SUVR in all SMC participants as a whole group, and in the SMCneg and SMCpos participants analyzed separately (Pearson's test; *p* < 0.05). Theta and alpha bands were defined on an individual basis based on TF and IAFp. The gamma band was fixed from 36 to 44 Hz.

**FIGURE 4:**
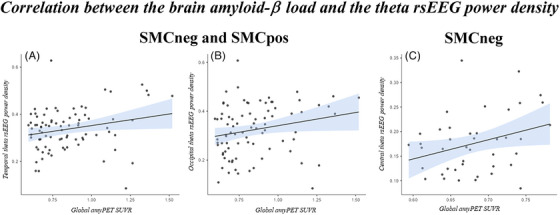
Correlation between the brain amyloid‐β load quantified by SUVR PET and the baseline rsEEG power density at the theta frequencies. The statistically significant results of an exploratory control analysis (Pearson's test, *p* < 0.05 uncorrected), testing the correlation between the brain amyloid load quantified by SUVR PET and the rsEEG‐theta power density at temporal, occipital, and central scalp regions of interest at the baseline (M0) in all SMC participants as a whole group (A and B) and in the SMCneg participants (C). No statistically significant results were observed in the SMCpos participants. amyPET SUVR, amyloid‐β load quantified by positron emission tomography standard uptake value ratio; rsEEG, resting‐state electroencephalographic; SMC, subjective memory complaint negative (SMCneg) or positive (SMCpos) to amyloid load; SUVR, standardized uptake value ratio.

Concerning the ANOVAs, no statistically significant three‐way Group × Education × ROI interaction (*p* > 0.05) was observed for the rsEEG theta/alpha 2 and for theta/alpha 3 power density. We observed a moderately significant correlation (Pearson's test) for the occipital (*r* = 0.22, *p* < 0.05) and temporal (*r* = 0.24, *p* < 0.05) rsEEG theta power density and the global SUVR by considering all SMC participants as a whole group (Figure 4A and B). By considering the different sub‐groups, we observed a statistically significant correlation was found between central theta (*r* = 0.32, *p* < 0.05; Figure 4C) and the global SUVR only for the SMCneg participants while no effect may be appreciated for the SMCpos participants (*p* > 0.05).

## 4 DISCUSSION

This INSIGHT study revealed that PET‐measured Alzheimer's brain amyloid load in SMC participants (SMCpos) did not lead to progressive cortical neurodegeneration from standard sMRI markers, alterations in rsEEG rhythms, or cognitive decline over 2 years.

In previous studies in SMC people, Alzheimer's amyloid load has been considered as a preclinical AD stage and a critical period for interventions aimed at preventing disease progression and the onset of objective cognitive deficits.^2^, ^54^, ^55^, ^56^ The present results suggest that the assumption of a direct association between brain amyloidosis and rapid cognitive decline in SMCpos adults merits more research. Along this line, some previous studies have reported only a weak correlation between mean brain amyloidosis and cognitive performance in cognitively unimpaired older adults.^2^, ^54^, ^55^, ^56^ Furthermore, other studies have failed to demonstrate a strong direct link between brain amyloidosis and cognitive decline over approximately 3 years,^58^ with similar findings reported in cognitively healthy older adults over periods of 2.5–7 years^58^, ^59^, ^60^.

Our results also do not support an association between brain amyloidosis “per se” and rapid cortical neurodegeneration in SMCpos participants over 2 years, when the educational attainment effect is not accounted for. This is consistent with prior neuroimaging studies, which have shown that brain amyloidosis in cognitively unimpaired older individuals is linked to structural brain changes over about 5 years, often driven in part by non‐AD pathologies.^61^, ^62^, ^63^ Conflicting findings on cortical neurodegeneration have been reported in ADMCI and ADD patients. On the one hand, amyloid deposition in the temporal lobe was associated with orbitofrontal cortical thinning over 2 years in ADMCI patients.^65^ On the other hand, only interactions between brain amyloidosis and vascular risk factors predicted brain atrophy and cognitive decline over 6 years in ADMCI patients.^66^ Interestingly, brain tauopathy, rather than amyloidosis, was a stronger predictor of cognitive decline and longitudinal atrophy over 2 years in ADMCI and mild ADD patients,^67^ while amyloidosis predicted faster parietal cortical neurodegeneration only in early‐onset ADD patients, not late‐onset cases.^68^

In the same line, we did not find an association between brain amyloidosis “per se” and progressive abnormalities in rsEEG rhythms in SMCpos participants over 2 years, when the educational attainment effect is not accounted for. While a previous INSIGHT‐preAD cohort study reported increased theta activity in mid‐frontal and posterior cingulate rsEEG sources at baseline and 2 years, respectively, the combination of rsEEG data from eyes‐closed and eyes‐open conditions complicated the interpretation due to mixed vigilance regulation and the presence of eye‐movement artifacts in the theta range.^69^ Another study observed rsEEG slowing and increased risk of progression from SMC to MCI over 2 years, but only when other parallel disease factors, such as CSF tau and medial lobe atrophy, were included in the analysis.^35^ Over longer periods of 7–9 years, rsEEG slowing has been shown to predict progression from subjective to objective cognitive deficits in individuals with unknown Aβ status^36^.

Collectively, these findings suggest that the relationship between brain amyloidosis and disease progression in SMCpos individuals may depend on the presence of multiple interacting dementia risk factors and may only become apparent over longer observational periods. Although Aβ is a core biomarker of preclinical Alzheimer's pathology, a more definitive assessment of this stage requires evaluating tau pathology and the synergistic toxic effects of Aβ and tau, also in relation to the evaluation of the clinical AD progression pathway.

Despite the absence of progressive abnormalities in rsEEG rhythms, an interesting discussion was raised about the present significant correlation between the occipital and temporal rsEEG theta rhythms and the brain amyloidosis in all the SMC participants. Indeed, it can be speculated that rsEEG slowing, that is, the enhanced rsEEG rhythms at < 7 Hz, which are thought to reflect underlying neurological dysfunction, may emerge prior to the Aβ‐positive stage in earlier stages of AD. Along this line, previous studies have shown a positive association between posterior rsEEG theta rhythms and regional amyloid deposition in patients with MCI.^70^ Similar results were observed for the association between the resting‐state magnetoencephalographic theta rhythms and the amyloid deposition evaluated in both AD‐relevant regions and globally.^71^ Abnormalities in ongoing EEG theta rhythms have been reported to be associated with hippocampal amyloid deposition in animal models.^72^ Notably, the linear association between rsEEG theta rhythms and brain amyloidosis was also observed in the only SMCneg group, but not in the SMCpos group, in the present study. On one hand, this could be related to nonlinear and complex association effects between brain amyloidosis and abnormal theta rhythms in preclinical AD phases .^73^ On the other hand, rsEEG theta rhythms may be sensitive to either sub‐threshold levels of Aβ deposition in SMC patients with early preclinical AD or other cerebral co‐pathologies. Future research requiring larger samples, clinical follow‐ups of more than 2 years, and more sophisticated statistical models are needed to explore these phenomena.

Our study further revealed that SMCneg participants with high educational attainment demonstrated the highest posterior rsEEG alpha rhythms over 2 years, indicative of stable neuroprotective effects. This suggests that high educational attainment exerts a stable neuroprotective influence on neurophysiological oscillatory mechanisms regulating dominant rsEEG alpha rhythms in SMC individuals who are not yet challenged by Alzheimer's pathology. In contrast, SMCpos participants with high educational attainment exhibited stable but significant reductions in posterior rsEEG alpha rhythms and parietal cortical thickness over 2 years, consistent with the compensatory effects of schooling. As posterior rsEEG alpha rhythms are linked to vigilance regulation, these findings highlight compensatory mechanisms that may protect cognitive status despite greater dysfunctions in neuromodulatory subcortical ascending systems and thalamocortical loops involved in arousal and vigilance regulation.^30^

Two key speculations arise from these findings. First, the lack of cognitive impairment in SMCpos participants with high educational attainment, despite altered rsEEG alpha rhythms, may reflect compensatory network recruitment strategies across different spatial scales. Enhanced neurogenesis and synaptic plasticity at smaller spatial scales may mitigate neuropathological processes such as neocortical amyloid plaques and cerebral amyloid angiopathy,^2^, ^3^, ^4^, ^5^, ^6^, ^15^, ^27^ potentially improving neurotransmission in cortical networks supporting cognition and episodic memory, including the frontal, default mode, and dorsal frontoparietal attention networks.^74^ Second, the stable abnormality of rsEEG alpha rhythms in SMCpos participants with high educational attainment suggests that vigilance dysfunctions (e.g., diurnal drowsiness, mental fatigue) may precede objective cognitive deficits. These findings underscore the value of home telemonitoring devices to track sleep–wake transitions and exaggerated daytime napping, as well as the potential utility of rsEEG measures as biomarkers for evaluating the efficacy of interventions targeting vigilance dysfunctions.

## 5 CONCLUSIONS

This monocentric study investigated whether SMC adults with substantial PET‐measured Alzheimer's brain amyloidosis (SMCpos) may exhibit signs of structural and neurophysiological impairment in the brain and cognitive decline over 2 years as a function of educational attainment.

The SMCpos participants with high educational attainment showed reduced posterior rsEEG alpha rhythms and parietal cortical thickness from structural MRIs compared to the SMCpos participants with low educational attainment, likely reflecting compensatory effects. Conversely, the SMCneg participants with high educational attainment showed greater posterior rsEEG alpha rhythms compared to their low‐attainment counterparts, suggesting neuroprotective effects. Importantly, these brain structural and neurophysiological measures remained stable over the 2‐year follow‐up period in older people with early Alzheimer's cerebral amyloid pathology. These results emphasize the importance of educational attainment as an important option for the prevention of AD in public health policies and for planning early intervention strategies.

In the evaluation of the present findings, some methodological remarks should be considered. The Alzheimer's brain tau pathology was not systematically assessed in the SMC participants, so the interpretation of the present longitudinal findings is limited to the interaction between educational attainment and amyloid‐related preclinical Alzheimer's pathology rather than targeting an overt preclinical AD status. Furthermore, educational attainment should be considered as a limited proxy of CR, as it does not formally account for quantitatively and qualitatively for aspects of lifelong intellectual, cultural, social, and leisure activities.

The present findings encourage further investments in future multicentric (harmonized) studies carried out in SMC participants, including a systematic evaluation of AD‐related amyloid and tau pathology, an extended assessment of CR beyond educational attainment, the above and structural (MRI) and neurophysiological (rsEEG) measures, and annual follow‐ups for more than 2 years to track the transition from earlier Alzheimer's pathology to preclinical and prodromal stages of AD. The results would provide a comprehensive neural and clinical model of the earlier AD progression, important for prevention and disease‐modifying intervention according to the precision medicine approach.^75^

## CONFLICT OF INTEREST STATEMENT

None of the authors have potential conflicts of interest or competing financial interests related to the present article. Their contribution to this article reflects only and exclusively their academic expertise. Some of them served or are serving as board or ad‐hoc reviewers, chief, associate, or handling editors for scientific journals. They disclaim any editorial interference with the editorial processing of the present article. Furthermore, some of them are inventors or co‐inventors of patents in the biomedical field and received honoraria as consultants from industries, pharmaceutical companies, or small‐medium enterprises, but this is irrelevant to the contents of the present article. B.D. has received consultancy fees from Biogen, Boehringer Ingelheim, Eli Lilly, and MedAvante and grants for his institution from Merck, Pfizer, and Roche. H.Ba. has received speaker fees from Roche. G.G. has received grants from France Alzheimer. M.‐C.P. has received grants from Fondation Vaincre Alzheimer, Laboratoires Servier, Pfizer, and Roche. H.H. is an employee of Eisai and his contribution to the present study and article reflects only and exclusively his academic expertise and own scientific opinions. This study has been initiated and prepared as part of an academic position at Sorbonne University, Paris, France. H.H. holds patents for in‐vitro determination methods (8916388, 20100062463, 7547553, and 20080199966) and in‐vitro procedures (8298784, 20100035286, and 20090263822), for diagnosis and early diagnosis of neurodegenerative disorders, for neurodegenerative markers for psychiatric conditions (20120196300 and 20080131921), and for a CSF diagnostic in‐vitro method for diagnosis of dementias and neuroinflammatory diseases (20080206797, 10921330). He serves as Reviewing Editor and previously as Senior Associate Editor for the journal Alzheimer's & Dementia, the journal of the Alzheimer's Association. A.V. declares no competing financial interests related to the present study and article. A.V.’s contribution to the present study and article reflects only and exclusively his academic expertise and own scientific opinions. This study has been initiated and prepared as part of an academic position at Sorbonne University, Paris, France. Since 2019, A.V. has collaborated with or worked for pharmaceutical and biotech companies (Roche, Eisai, MagQu, Teva, Angelini, Servier). Author disclosures are available in the Supporting Information.

## COLLABORATORS

(INveStIGation of AlzHeimer's PredicTors in subjective memory complainers) INSIGHT‐preAD study group: Hovagim Bakardjian, Habib Benali, Hugo Bertin, Joel Bonheur, Laurie Boukadida, Nadia Boukerrou, Enrica Cavedo, Patrizia Chiesa, Olivier Colliot, Bruno Dubois, Marion Dubois, Stéphane Epelbaum, Geoffroy Gagliardi, Remy Genthon, Marie‐Odile Habert, Harald Hampel, Marion Houot, Aurélie Kas, Foudil Lamari, Marcel Levy, Simone Lista, Christiane Metzinger, Fanny Mochel, Francis Nyasse, Catherine Poisson, Marie‐Claude Potier, Marie Revillon, Antonio Santos, Katia Santos Andrade, Marine Sole, Mohmed Surtee, Michel Thiebaud de Schotten, Andrea Vergallo, and Nadjia Younsi.

## CONSENT STATEMENT

All experiments were performed with each participant or caregiver's informed and overt consent, per the Code of Ethics of the World Medical Association (Declaration of Helsinki) and the standards established by the local Institutional Review Board at the participating center (Ethical approval number: 2013‐Fev‐13150). All participants or their representatives gave written informed consent for the use of their clinical data for research purposes. Participants were recruited without discrimination based on gender, ethnicity, socioeconomic status, or other personal characteristics, ensuring a diverse and representative sample.

## Supporting information



Supporting Information

Supporting Information
