# Supplementary material for: Educational attainment, electroencephalographic rhythms, cortical structure, and cognitive performance over 2 years in older adults with subjective memory complaints and brain amyloidosis
Source: Alzheimers Dement. 2025 Jul 14;21(7):e70438. doi: 10.1002/alz.70438 (PMC12260118; doi:10.1002/alz.70438)
Supplement: Supplementary file 2 — Supporting Information [file ALZ-21-e70438-s002.docx]

***Supplementary Materials***

**Educational attainment, electroencephalographic rhythms, cortical structure, and cognitive performance over 2 years in older adults with subjective memory complaints and brain amyloidosis**

Susanna Lopez^1^, Harald Hampel^2$^, Claudio Del Percio^1^, Giuseppe Noce^3^, Roberta Lizio^1,4^, Stefan J. Teipel^5,6^, Martin Dyrba^5^, Gabriel González-Escamilla^7^, Hovagim Bakardjian^8,9^, Patrizia Andrea Chiesa^2,8,9^, Enrica Cavedo^2,10,11^, Andrea Vergallo^2^ , Pablo Lemercier^2,10,11^, Giuseppe Spinelli^10,11^, Michel J. Grothe^6^, Marie-Claude Potier^9^, Fabrizio Stocchi^12,13^, Chiara Coletti^12^, Raffaele Ferri^4^, Matteo Pardini^14,15^, Marie-Odile Habert^8,9,16^, Simone Marziali^17^, Bruno Dubois^8,9^, Claudio Babiloni^1,17$^, and INSIGHT-preAD study group

 $ = Equally contributing Authors

*1 Department of Physiology and Pharmacology "Erspamer", Sapienza University of Rome, P.le Aldo Moro, 5, 00185, Rome, Italy;*

*2 Sorbonne Université, Alzheimer Precision Medicine (APM), AP-HP, Pitié-Salpêtrière Hospital, Boulevard de l'hôpital, F-75013, Paris, France;*

*3 IRCCS Synlab SDN, Via Emanuele Gianturco, 113, 80143, Naples, Italy;*

*4 Oasi Research Institute - IRCCS, Via Conte Ruggero, 73, 94018, Troina, Italy;*

*5 Department of Psychosomatic Medicine, University of Rostock, Gehlsheimer Str. 20 18147, Rostock, Germany;*

*6 German Center for Neurodegenerative Diseases (DZNE) – Gehlsheimer Str. 20 18147, Rostock, Germany;*

*7 Department of Neurology, Saarland University,* *Gebäude 90, Kirrberger Straße, 66421, Homburg, Germany;*

*8 Institute of Memory and Alzheimer's Disease (IM2A), Department of Neurology, Pitié-Salpêtrière Hospital, AP-HP, Boulevard de l'hôpital, F-75013, Paris, France;*

*9 Institut du Cerveau et de la Moelle épinière, ICM, INSERM U1127, CNRS UMR 7225, Sorbonne Université, F- 75013, Paris, France;*

*10 Centre pour l'Acquisition et le Traitement des Images, (CATI platform),*[*www.cati-neuroimaging.com*](http://www.cati-neuroimaging.com/)*;*

*11 Laboratoire d'Imagerie Biomédicale, CNRS, INSERM, Sorbonne Université, LIB, F-75006, Paris, France;*

*12 IRCCS San Raffaele, Via della Pisana, 235, 00163, Rome, Italy;*

*13 Telematic University, San Raffaele, Via di Val Cannuta, 247, 00166, Rome, Italy;*

*14 Dipartimento di Neuroscienze, Oftalmologia, Genetica, Riabilitazione e Scienze Materno-infantili (DiNOGMI), Università di Genova,* *Largo Paolo Daneo, 3, 16132 Genova, Italy;*

*15 Neurofisiopatologia, IRCCS Ospedale Policlinico San Martino, Largo R. Benzi, 10, 16132, Genova, Italy*

*16 AP-HP, Pitié-Salpêtrière Hospital, Department of Nuclear Medicine, F-75013, Paris, France;*

*17 Hospital San Raffaele Cassino, Via Gaetano di Biasio, 1, 03043 Cassino, Italy.*

Corresponding author: Prof. Claudio Babiloni

Department of Physiology and Pharmacology "V. Erspamer"

University of Rome "La Sapienza"

P. le A. Moro 5, 00185, Rome, Italy

Phone: +39 0649910989

E-mail: [claudio.babiloni@uniroma1.it](mailto:claudio.babiloni@uniroma1.it)

**Running title:** Longitudinal effect of education and preclinical amyloidosis on resting-state alpha rhythms.

**Keywords:** Resting state EEG alpha rhythms; Preclinical Alzheimer’s disease (AD) amyloidosis; Subjective Memory Complaint (SMC); Magnetic Resonance Imaging (MRI); INSIGHT-Pre-AD study.

***1. Supplementary Material Methods***

*1.1 Regions of Interest (ROIs) including rsEEG electrodes*

Five regions of interest (ROIs) were used considering the frontal, central, parietal, occipital, and temporal electrodes of the 10-10 electrode montage system. Specifically, these ROIs included the electrodes, as reported in Table Supplementary Material 1 (SM1).

| **Electrodes** | **Region of Interest (ROI)** |
| --- | --- |
| AF7, AF3, AFz, AF4, AF8, FP1; FPz; FP2; F10; F9; F7; F5; F3; F1; Fz; F2; F4; F6; F8; FC5; FC3; FC1; FCz; FC2; FC4; FC6 | Frontal |
| C5, C3, C1, C2, C4, C6, CP5, Cp3, CP1, CPz, Cp2, CP4, CP6 | Central |
| P9, P5, P3, P1, Pz, P2, P4, P6, P10, PO7, PO3, POz, PO4, PO8 | Parietal |
| O1, Oz, O2 | Occipital |
| FT9, FT7, FT8, FT10, T5-P7, T6-P8, T3-T7, T4-T8, T9, TP7, TP8, T10 | Temporal |

**Table Supplementary Materials 1 (SM1).** *Regions of Interest (ROIs) including resting state eyes-closed electroencephalographic (rsEEG) electrodes.* Grouping of 68 scalp electrodes for the definition of the Regions of Interest (ROIs) from the electrode montage used for the present recordings of resting state eyes-closed electroencephalographic (rsEEG) activity. The rsEEG results of the present study were based on the analysis performed from those 68 scalp electrodes of the 10-10 montage system.

The electrode montage is depicted in Figure SM1.

**
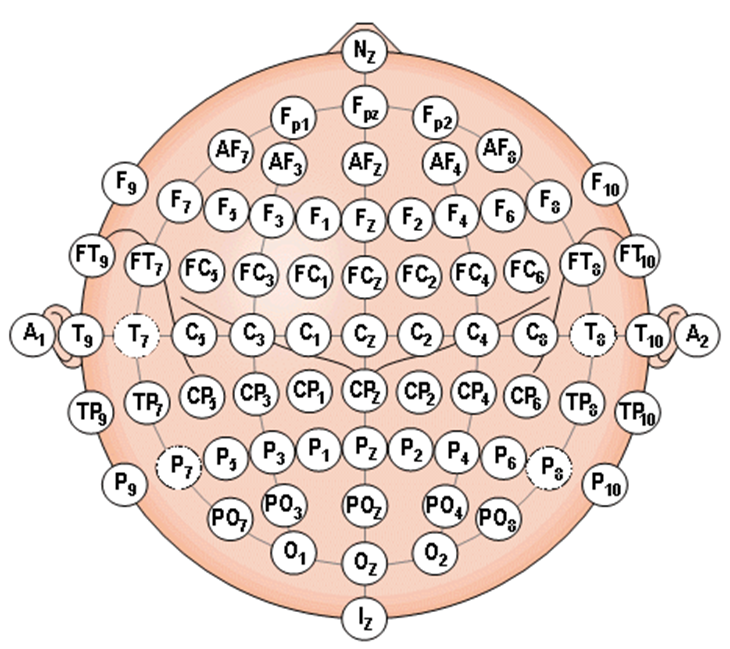
**

**Figure Supplementary Materials 1 (SM1).** *Standard electrode 10-10 montage.*

*1.2 Individual frequency bands definition*

The rsEEG frequency bands of interest were individually determined based on the following frequency landmarks: the transition frequency (TF) and the IAFp [53, 54]. The TF marks the transition frequency between the theta and alpha bands and is defined as the frequency showing the minimum rsEEG power density between 3 Hz and 8 Hz (i.e., between the delta and the alpha power peak). The IAFp is defined as the frequency showing a maximum rsEEG power density peak between 6 Hz and 14 Hz. The analysis of the TF and IAFp was performed on rsEEG power density averaged across Fz, Cz, Pz, O1, and O2 scalp electrodes, which typically show EEG delta, theta, and alpha rhythms with low biological noise (e.g., the scalp midline is relatively far from temporal and frontal muscles) and optimal electrical contacts using a standard EEG helmet [40, 41].

In detail, the individual frequency bands from delta to alpha were determined as follows, from rsEEG power density spectra computed with a frequency resolution of 0.5 Hz:

- Delta from TF -4 Hz to TF -2 Hz.
- Theta from TF -2 Hz to TF.
- Alpha 1 from TF to the midpoint of the TF-IAFp range.
- Alpha 2 from the middle of the TF-IAFp range to IAFp.
- Alpha 3 from IAFp to IAFp + 2 Hz.

An example of the rsEEG power density with the indication of the TF, IAFp, and the individual frequency band I illustrated in Figure SM2.

**
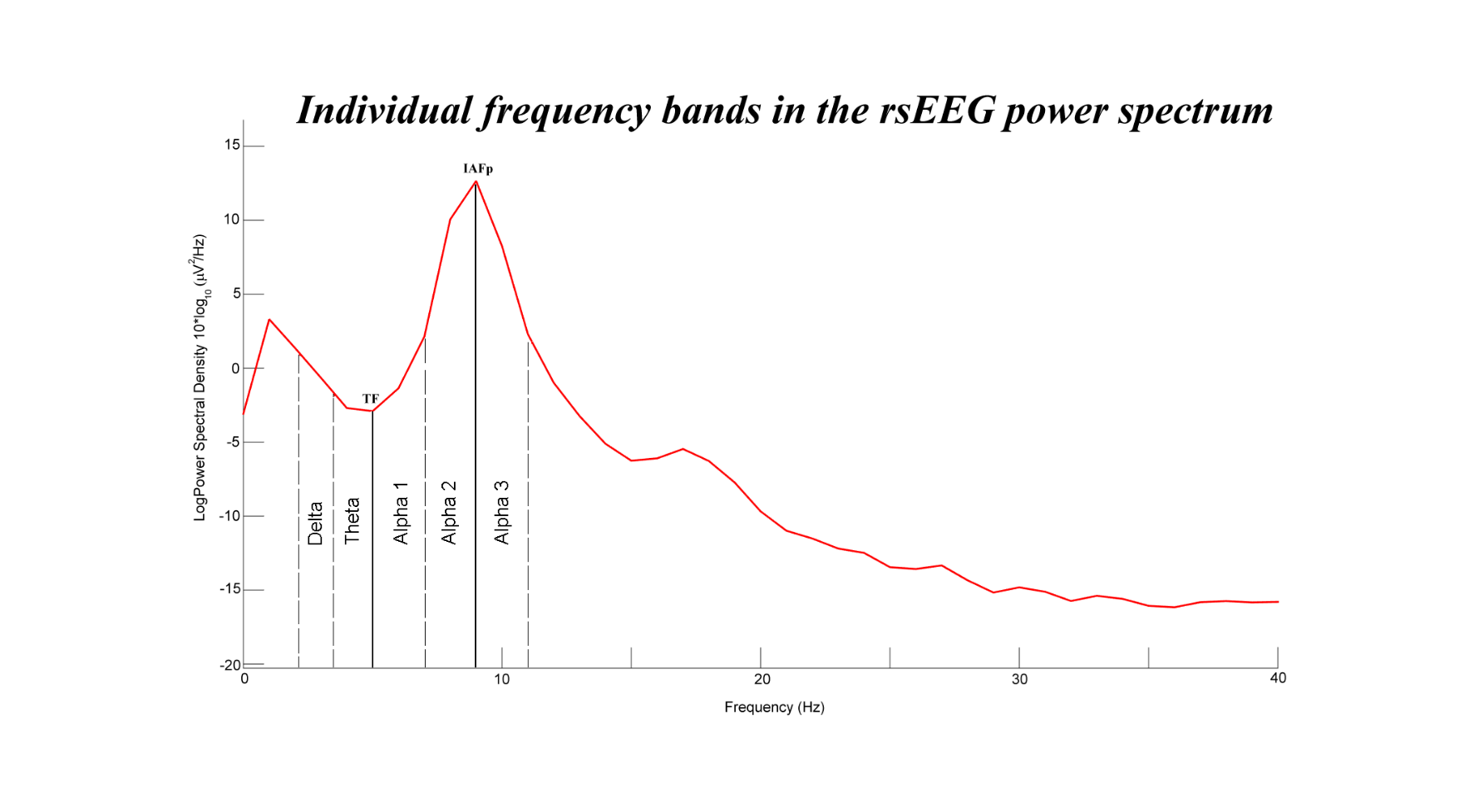
**

**Figure SM2.** *Individual frequency bands definition.* rsEEG frequency bands of interest were individually determined based on the following frequency landmarks: the transition frequency (TF) and the Individual Alpha Frequency peak (IAFp).

***2. Supplementary Material Results***

*2.1 Neurophysiological TF and IAFp hallmarks*

At the baseline (M0), the mean TF was 5.8 Hz (± 0.2 standard error of the mean, SE) in the SMCneg Edu-, 5.5 Hz (± 0.2 SE) in the SMCneg Edu+, 5.4 Hz (± 0.1 SE) in the SMCpos Edu-, and 5.4 Hz (± 0.1 SE) in the SMCpos Edu+ sub-groups. The mean IAFp was 9.8 Hz (± 0.2 SE) in the SMCneg Edu-, 9.5 Hz (± 0.2 SE) in the SMCneg Edu+, 9.1 Hz (± 0.1 SE) in the SMCpos Edu-, and 9.1 Hz (± 0.1 SE) in the SMCpos Edu+ sub-groups. At the 12-month follow-up (M12), the mean TF was 5.5 Hz (± 0.2 SE) in the SMCneg Edu-, 5.7 Hz (± 0.2 SE) in the SMCneg Edu+, 5.2 Hz (± 0.1 SE) in the SMCpos Edu-, and 5.1 Hz (± 0.2 SE) in the SMCpos Edu+ sub-groups. The mean IAFp was 9.4 Hz (± 0.2 SE) in the SMCneg Edu-, 9.3 Hz (± 0.2 SE) in the SMCneg Edu+, 9.2 Hz (± 0.1 SE) in the SMCpos Edu-, 9.1 Hz (± 0.1 SE) in the SMCpos Edu+ sub-groups. At the 24-month follow-up (M24), the mean TF was 5.7 Hz (± 0.2 SE) in the SMCneg Edu-, 5.4 Hz (± 0.2 SE) in the SMCneg Edu+, 5.4 Hz (± 0.1 SE) in the SMCpos Edu-, and 5.4 Hz (± 0.1 SE) in the SMCpos Edu+ sub-groups. The mean IAF was 9.2 Hz (± 0.2 SE) in the SMCneg Edu-, 9.2 Hz (± 0.2 SE) in the SMCneg Edu+, 9.1 Hz (± 0.3 SE) in the SMCpos Edu-, and 9.0 Hz (± 0.2 SE) in the SMCpos Edu+ sub-groups.

*2.2 Longitudinal comparisons of the amyloidosis and brain integrity between the SMCneg and SMCpos groups and effect of educational attainment*

No statistically significant effect (p > 0.05) was observed concerning the 3-way Time (M0, M24) × Group (SMCneg and SMCpos) × Education (Edu- and Edu+) interaction for any of the amyloid PET and MRI variables analyzed.

The results of the not significant (p > 0.05) ANOVA models, in which no interaction with the Education factor over the M0 and M24 timepoints was observed, are illustrated in Figure SM3. Mostly, the Group effect was statistically significant (p < 0.05), suggesting that SMCpos participants were characterized by higher amyloid deposition in the brain and higher neurodegeneration both at M0 and M24. There was a trend towards the higher neurodegeneration (lower cortical volume and cortical thinning) in several posterior regions, as well as higher white matter hyperintensity in the SMCpos Edu+ sub-group as compared to the SMCpos Edu- and SMCneg sub-groups.

**
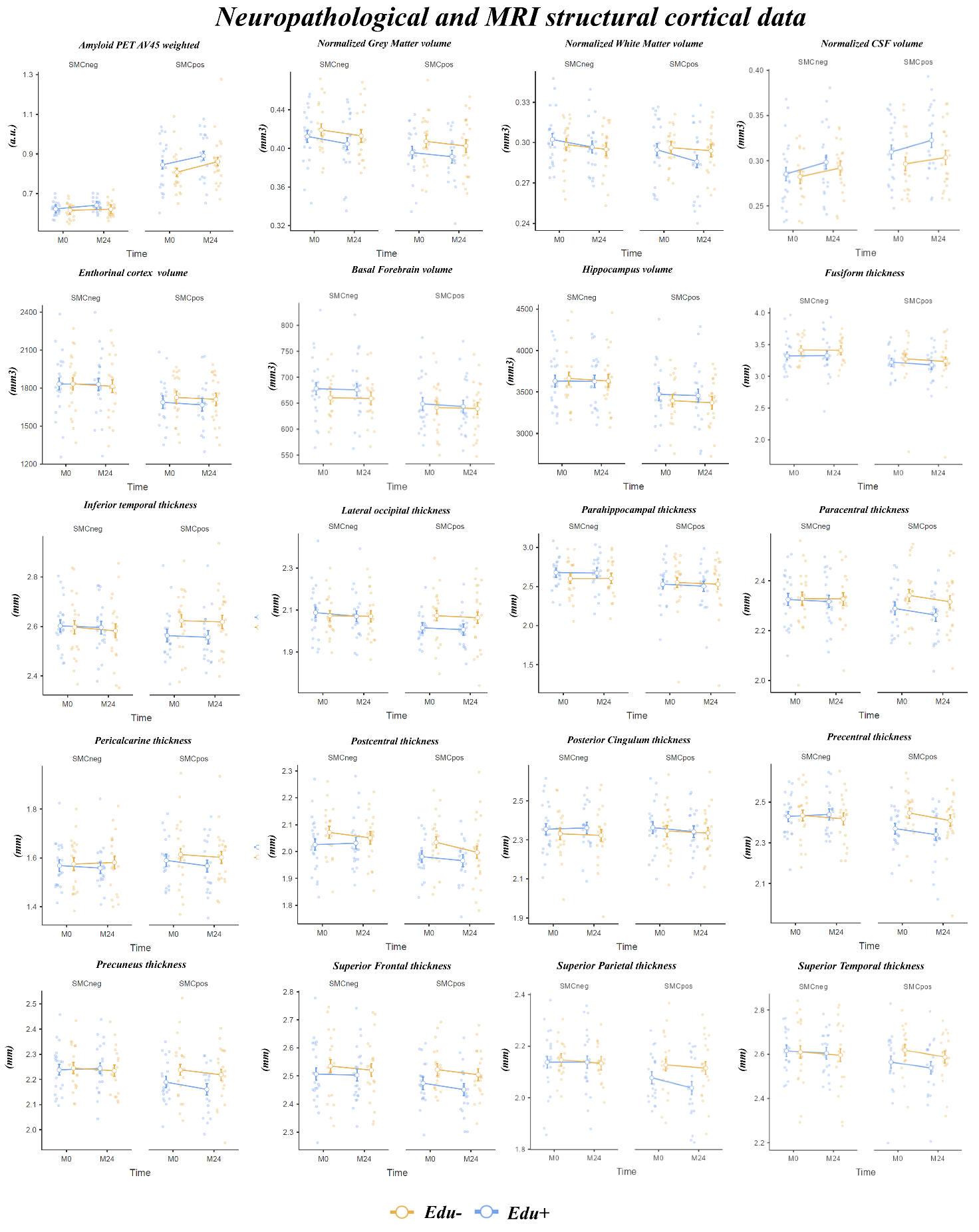
**

**Figure SM3.** *Neuroimaging (amyloid PET and MRI) data in the SMC seniors at the M0 and M24 timepoi*nts. Descriptive graphs illustrate the mean value (± standard error mean, SE) of the neuroimaging (amyloid PET and MRI) data in the SMCneg and SMCpos Edu- and Edu+ sub-groups at the baseline (M0) and 24-month follow-up (M24). No statistically significant effect (p > 0.05) was observed concerning the 3-way Time (M0, M24) × Group (SMCneg and SMCpos) × Education (Edu- and Edu+) interaction for any of the amyloid PET and MRI variables analyzed. *Legend: AV45 = ^18^F-florbetapir* *amyloid positron emission tomography; MRI = magnetic resonance imaging; SMC = subjective memory complaint negative (SMCneg) or positive (SMCpos) to amyloid load; M0 = baseline acquisition; M24 = 24-month follow-up acquisition.*

*2.3 Longitudinal comparisons in the cognitive-functional abilities between the SMCneg and SMCpos groups and effect of educational attainment*

No statistically significant effect (p > 0.05) was observed concerning the 3-way Time (M0, M12, M24) × Group (SMCneg and SMCpos) × Education (Edu- and Edu+) interaction for any of the neuropsychological variables analyzed.

The results of the not significant (p > 0.05) ANOVA models, in which no interaction with the Education factor over the M0, M12, and M24 timepoints was observed, are illustrated in Figure SM4. Mostly, the Group effect was statistically significant (p < 0.05) in the DMS 48 learning [1], Copy of the Rey figure [2] immediate (time), Copy of the Rey figure after 3 minutes (time), Copy of the Rey figure after 30 minutes, suggesting that SMCpos participants were characterized by higher visual recognition impairment as compared to SMCneg seniors at both M0, M12, and M24 but without a clear progression over 2 years.

**
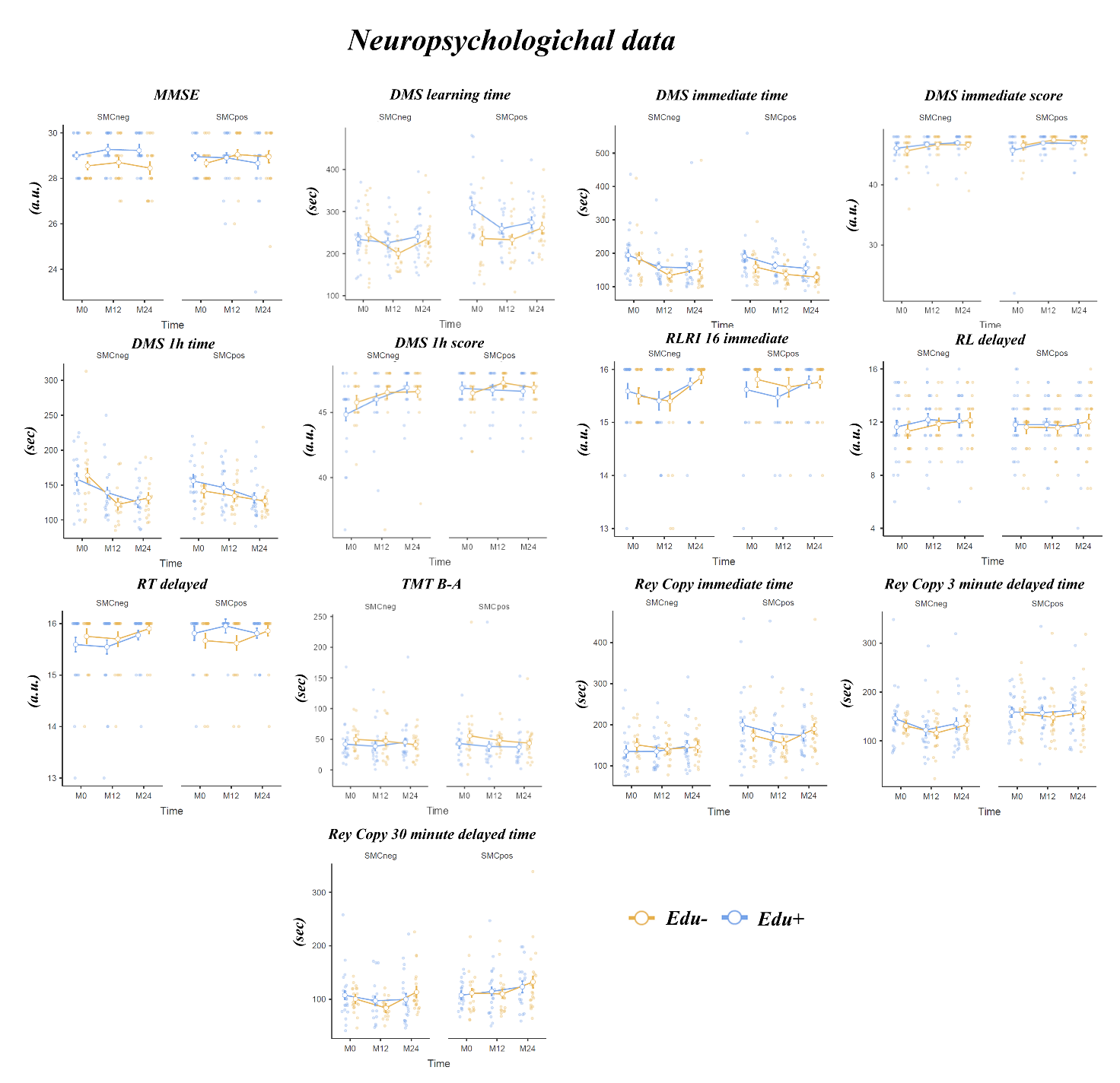
**

**Figure SM4.** *Neuropsychological data in the SMC seniors at the M0, M12, and M24 timepoi*nts. Descriptive graphs illustrate the mean value (± standard error mean, SE) of the neuropsychological data in the SMCneg and SMCpos Edu- and Edu+ sub-groups at the baseline (M0), 12-month follow-up (M12) and 24-month follow-up (M24). No statistically significant effect (p > 0.05) was observed concerning the 3-way Time (M0, M24) × Group (SMCneg and SMCpos) × Education (Edu- and Edu+) interaction for any of the neuropsychological variables analyzed. *Legend: MMSE = Mini-Mental State Examination; TMT = Trial Making test B-A; SMC = subjective memory complaint negative (SMCneg) or positive (SMCpos) to amyloid load; M0 = baseline acquisition; M12 = 12-month follow-up acquisition; M24 = 24-month follow-up acquisition.*

*2.4 Control analysis on the association between rsEEG alpha rhythms and amyloidosis/brain integrity (amyloid PET and MRI markers)*

For cross-validation purposes about the link between the neuroimaging data (amyloidosis as revealed by amyloid PET and the brain integrity revealed by structural MRI measures) and the neurophysiological signature indexed by the posterior rsEEG alpha rhythms, we performed a general linear regression (GLR) analysis at M0 and M24 timepoints.

More specifically, the first (control) cross-validation analyses included several linear regression models (one model for each rsEEG and the corresponding neuroimaging variable at M0 and M24) having the following features:

- Dependent rsEEG variables: parietal and occipital rsEEG alpha 2 and alpha 3 power density (one model for each variable).
- Predictors: Group (SMCneg, SMCpos), Education (Edu+, Edu-), neuroimaging variable (amyloid deposition measured by PET, normalized grey matter volume, normalized white matter volume, normalized CSF volume, basal forebrain volume, hippocampus volume, entorhinal cortex volume, entorhinal cortex thickness, fusiform thickness, inferior parietal thickness, inferior temporal thickness, lateral occipital thickness, middle temporal thickness, para-hippocampal thickness, paracentral thickness, pericalcarine thickness, postcentral thickness, posterior cingulate thickness, precentral thickness, precuneus thickness, superior frontal thickness, superior parietal thickness, superior temporal thickness, insula thickness, white matter hyperintensities; one model for each variable), 2 and 3-way Group × Education × MRI variable interactions.

The results showed not statistically significant 3-way Group × Education × neuroimaging (amyloid PET or MRI) variable interaction, both at M0 and M24. There were only statistically significant main and 2-way Group × basal forebrain volume interaction, both at M0 and M24, in predicting the parietal and occipital rsEEG alpha 2 and alpha 3 power density (see Table SM2 and SM3 for details).

| **Model** | **Predictors** | **β (± SE)** | **p-value** |
| --- | --- | --- | --- |
| **M0 A2 P ~ Group * Edu * M0 BF volume**  F = 0.067, p < 0.005  R^2 = 0.24, Adjusted R^2 = 0.17 | Group | 4.2383 ± 1.04 | 0.0001 |
|  | M0 BF volume | 0.0034 ± 0.0012 | 0.0061 |
|  | Group*M0 BF volume | -0.0063 ± 0.0016 | 0.0002 |
|  | Group*Edu*M0 BF volume | 0.0056 ± 0.0021 | 0.0087 |
| **M0 A2 O ~ Group * Edu * M0 BF volume**  F = 0.055, p < 0.01  R^2 = 0.22, Adjusted R^2 = 0.15 | Group | 4.6694 ± 1.2778 | 0.0005 |
|  | M0 BF volume | 0.0037 ± 0.0015 | 0.016 |
|  | Group*M0 BF volume | -0.0069 ± 0.002 | 0.0008 |
|  | Group*Edu*M0 BF volume | 0.0064 ± 0.0025 | 0.0145 |

**Table SM2.** *Association between the basal forebrain (BF) volume and the rsEEG-dependent variables of interest at the M0 timepoint.* Results of a control statistical session based on linear regression models (p < 0.05 uncorrected), testing the effect of the Group (SMCneg and SMCpos) and Edu (Edu- and Edu+) predictors on the association between the basal forebrain (BF) volume and the rsEEG-dependent variables of interest at the baseline (M0) acquisition. The only statistically significant results were observed for the parietal and occipital rsEEG alpha 2 power density (p < 0.05 uncorrected). *Legend: M0 = baseline acquisition; BF = basal forebrain; rsEEG = resting-state electroencephalographic; A2 P = parietal rsEEG alpha 2 power density; A2 O = occipital rsEEG alpha 2 power density.*

| **Model** | **Predictors** | **β (± SE)** | **p-value** |
| --- | --- | --- | --- |
| **M24 A2 P ~ Group * Edu * M24 BF volume**  F = 0.66, p < 0.005  R^2 = 0.24, Adjusted R^2 = 0.17 | Group | 3.4547 ± 1.078 | 0.002 |
|  | M24 BF volume | 0.0027 ± 0.0013 | 0.0331 |
|  | Group*M24 BF volume | -0.0052 ± 0.0017 | 0.0024 |
| **M24 A2 O ~ Group * Edu * M24 BF volume**  F = 0.29, p < 0.005  R^2 = 0.23, Adjusted R^2 = 0.17 | Group | 4.0827 ± 1.2764 | 0.002 |
|  | M24 BF volume | 0.0031 ± 0.0015 | 0.0431 |
|  | Group*M24 BF volume | -0.0061 ± 0.002 | 0.0026 |
| **M24 A3 P ~ Group * Edu * M24 BF volume**  F = 0.26, p < 0.05  R^2 = 0.18, Adjusted R^2 = 0.12 | Group | 3.1292 ± 1.0575 | 0.0041 |
|  | M24 BF volume | 0.0025 ± 0.0012 | 0.0486 |
|  | Group*M24 BF volume | -0.0047 ± 0.0016 | 0.005 |
| **M24 A3 O ~ Group * Edu * M24 BF volume**  F = 0.15, p < 0.05  R^2 = 0.20, Adjusted R^2 = 0.13 | Group | 4.0776 ± 1.2641 | 0.0019 |
|  | M24 BF volume | 0.0032 ± 0.0015 | 0.0311 |
|  | Group*M24 BF volume | -0.0061 ± 0.0019 | 0.0023 |

**Table SM3.** *Association between the basal forebrain (BF) volume and the rsEEG dependent variables of interest at the M24 timepoint.* Results of a control statistical session based on linear regression models (p < 0.05 uncorrected), testing the effect of the Group (SMCneg and SMCpos) and Edu (Edu- and Edu+) predictors on the association between the basal forebrain (BF) volume and the rsEEG-dependent variables of interest at the 24-month follow-up (M24) acquisition. The statistically significant results were observed for all the rsEEG power density variables considered, i.e., the posterior and occipital rsEEG alpha 2 and alpha 3 power density (p < 0.05 uncorrected). *Legend: M24 = 24-month follow-up acquisition; BF = basal forebrain; rsEEG = resting-state electroencephalographic; A2 P = parietal rsEEG alpha 2 power density; A2 O = occipital rsEEG alpha 2 power density; A3 P = parietal rsEEG alpha 3 power density; A3 O = occipital rsEEG alpha 3 power density.*

The post-hoc analysis revealed that, only in the SMCneg participants, there was a statistically significant (p < 0.05, FDR corrected) positive association between the basal forebrain volume and the parietal and occipital rsEEG alpha 2 power density at M0 (Figure SM5). The same patterns were observed at M24 (Figure SM6) for the parietal and occipital rsEEG alpha 2 and alpha3 power density.

**
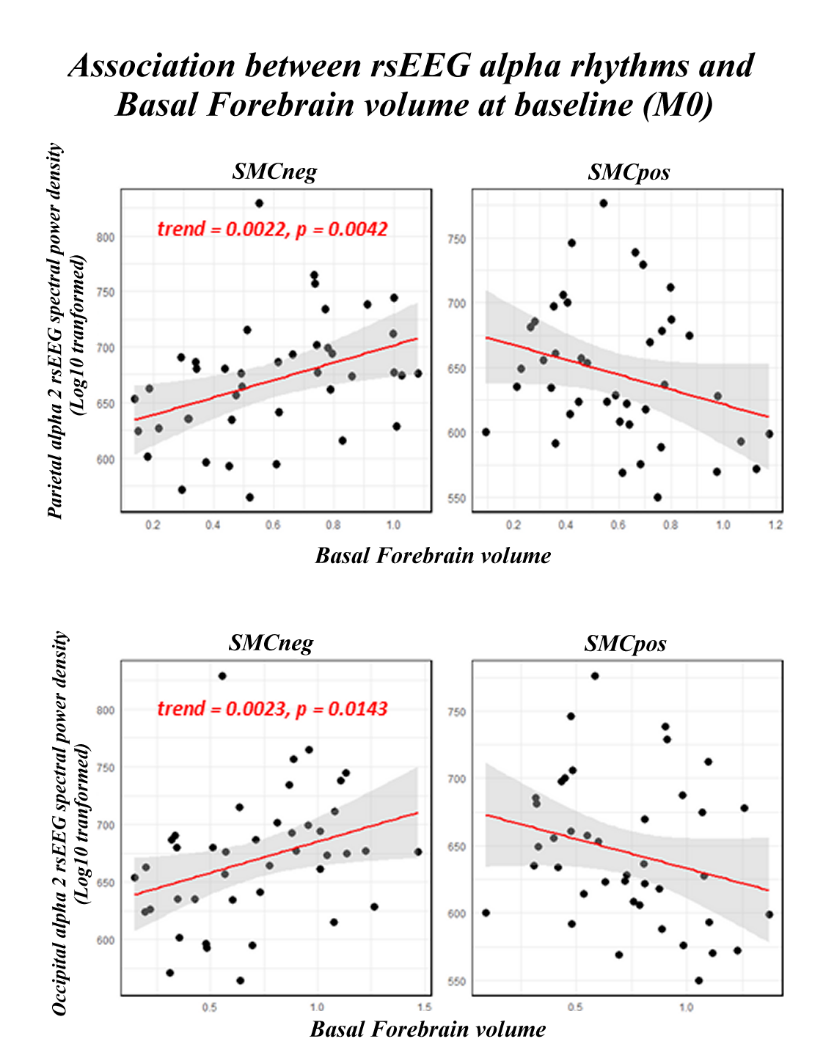
**

**Figure SM5.** *Association between the basal forebrain (BF) volume and the rsEEG-dependent variables of interest at the M0 timepoint.* Scatterplots of the parietal (upper) and occipital (lower) rsEEG alpha 2 power density (estimated marginal means by General Linear Regression model) in the SMCneg (left) and SMCpos (right) sub-groups expressing the association with the basal forebrain (BF) volume at the baseline (M0) acquisition. Inside each graph, the statistically significant associations calculated by the sub-group analysis using estimated marginal means are highlighted in red (p < 0.05; FDR corrected), as well as the slope coefficients and p-values. *Legend: SMC = subjective memory complaint negative (SMCneg) or positive (SMCpos) to amyloid load; M0 = baseline acquisition.*

**
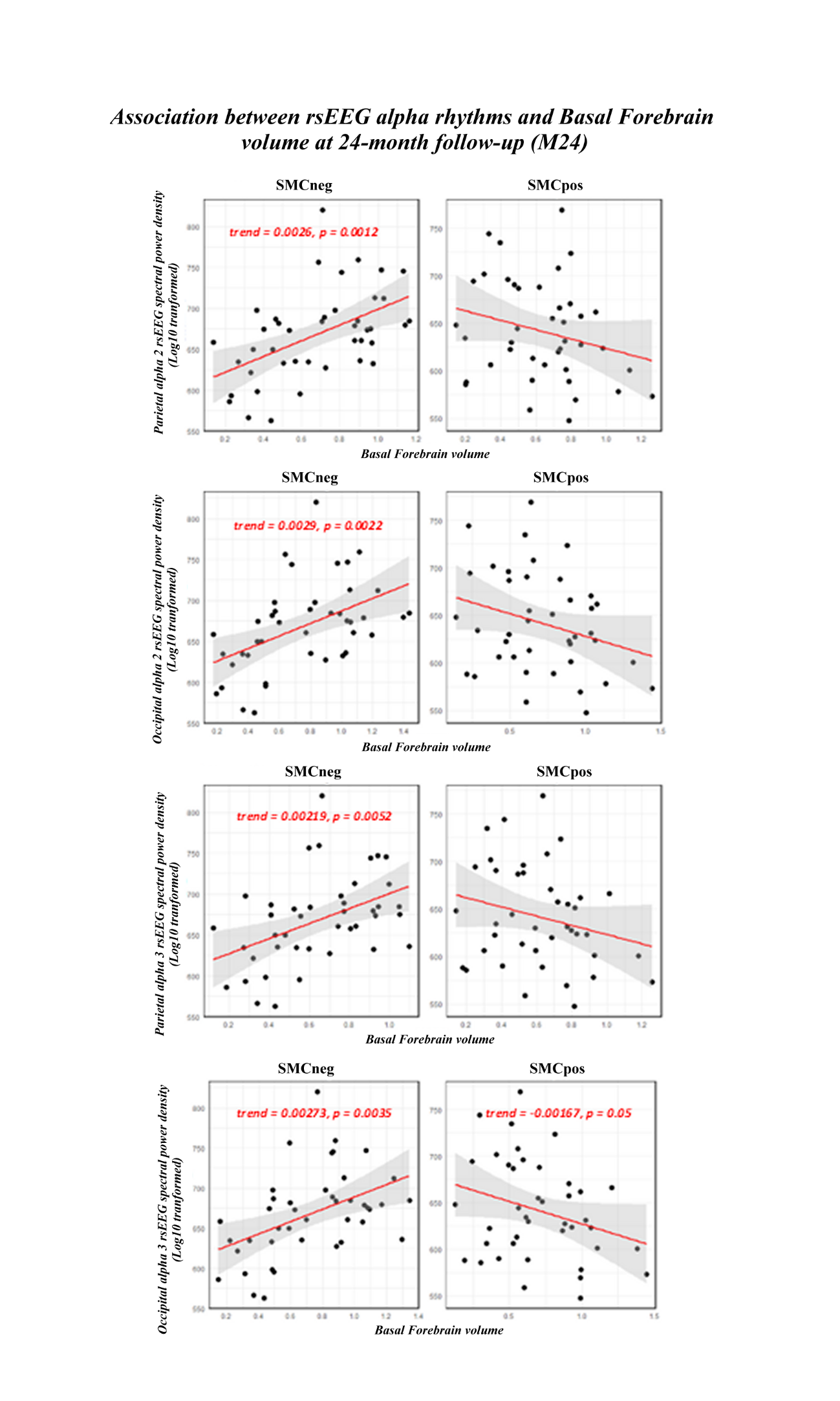
**

**Figure SM6.** *Association between the basal forebrain (BF) volume and the rsEEG-dependent variables of interest at the M24 timepoint.* Scatterplots of the parietal and occipital rsEEG alpha 2 (first and second row, respectively) and alpha 3 (third and fourth row, respectively) power density (estimated marginal means by General Linear Regression model) in the SMCneg (left) and SMCpos (right) sub-groups expressing the association with the basal forebrain (BF) volume at the 24-month follow-up (M24) acquisition. Inside each graph, the statistically significant associations calculated by the sub-group analysis using estimated marginal means are highlighted in red (p < 0.05; FDR corrected), as well as the slope coefficients and p-values. *Legend: SMC = subjective memory complaint negative (SMCneg) or positive (SMCpos) to amyloid load; M24 = 24-month follow-up acquisition.*

*2.5 Control analysis on the association between rsEEG alpha rhythms and cognitive-functional abilities*

For cross-validation purpose about the link between the neurophysiological signature indexed by the posterior rsEEG alpha rhythms, and the cognitive-functional abilities measured by neuropsychological tests, we performed a general linear regression (GLR) analysis at M0, M12, and M24 timepoints.

The second (control) cross-validation rsEEG analysis included several linear regression models (one model for each rsEEG and the corresponding neuropsychological score variable at M0, M12, and M24) having the following features:

• Dependent neuropsychological score: MMSE, DMS 48 learning, DMS 48 immediate recall (time), DMS 48 immediate recall score, DMS 48 1hour delayed (time), DMS 48 1hour delayed score, Trial Making test (TMT) B-A, Copy of the Rey figure immediate (time), Copy of the Rey figure after 3 minutes (time), Copy of the Rey figure after 30 minutes (time; one model for each variable);

• Predictors: Group (SMCneg, SMCpos), Education (Edu+, Edu-), rsEEG variables (parietal and occipital alpha 2 and alpha 3 power density; one model for each variable), 2 and 3-way Group × Education × rsEEG variable interactions.

The results showed statistically significant 3-way Group × Education × rsEEG variable interaction in predicting the DMS 48 1hour (time) and the Copy of the Rey figure immediate (time), both at M0 (Table SM4) and M12 (Table SM5).

| **Model** | **Predictors** | **β (± SE)** | **p-value** |
| --- | --- | --- | --- |
| **M0 Copy of the Rey figure immediate ~ Group * Edu * M0 A2 P**  F = 6.22, p < 0.01  R^2 = 0.20, Adjusted R^2 = 0.127 | Group*Edu | 205.4438 ± 76.3081 | 0.0087 |
|  | Group*Edu*M0 A2 P | -310.2675 ± 119.3783 | 0.0112 |
| **M0 DMS 48 1h time~ Group * Edu * M0 A2 O**  F = 10.41, p < 0.0001  R^2 = 0.36, Adjusted R^2 = 0.290 | Group | -57.7414 ± 28.6854 | 0.0481 |
|  | Edu | -78.4902 ± 30.8612 | 0.0133 |
|  | Group*Edu | 126.5407 ± 44.476 | 0.0059 |
|  | Edu*M0 A2 O | 87.4144 ± 39.4093 | 0.0299 |
|  | Group*Edu*M0 A2 O | -135.1685 ± 57.8256 | 0.0224 |
| **M0 Copy of the Rey figure immediate ~ Group * Edu * M0 A2 O**  F = 6.57, p < 0.01  R^2 = 0.24, Adjusted R^2 = 0.243 | Group*Edu | 256.7915 ± 74.8525 | 0.001 |
|  | Group*Edu*M0 A2 O | -324.7812 ± 96.3524 | 0.0012 |
| **M0 Copy of the Rey figure immediate ~ Group * Edu * M0 A3 P**  F = 6.12, p < 0.05  R^2 = 0.19, Adjusted R^2 = 0.112 | Group*Edu | 180.1343 ± 75.2433 | 0.0191 |
|  | Group*Edu*M0 A3 P | -272.9398 ± 121.5989 | 0.0277 |
| **M0 Copy of the Rey figure immediate ~ Group * Edu * M0 A3 O**  F = 6.12, p < 0.05  R^2 = 0.19, Adjusted R^2 = 0.112 | Group*Edu | 236.0623 ± 74.7008 | 0.0023 |
|  | Group*Edu*M0 A3 O | -297.7587 ± 97.191 | 0.003 |

**Table SM4.** *Association between the rsEEG variables and the neuropsychological dependent variables at the M0 timepoint.* Results of a control statistical session based on linear regression models (p < 0.05 uncorrected), testing the effect of the Group (SMCneg and SMCpos) and Edu (Edu- and Edu+) predictors on the association between the rsEEG variables of interest and the neuropsychological dependent variables at the baseline (M0) acquisition. The statistically significant results were observed for all the rsEEG power density variables considered, i.e., the posterior and occipital rsEEG alpha 2 and alpha 3 power density (p < 0.05 uncorrected). *Legend: M0 = baseline acquisition; Copy of the Rey figure immediate = duration of the immediate copy of the Rey Figure (s); DMS 48 1h score = DMS 48 recognition after 1hour score; rsEEG = resting-state electroencephalographic; A2 P = parietal rsEEG alpha 2 power density; A2 O = occipital rsEEG alpha 2 power density; A3 P = parietal rsEEG alpha 3 power density; A3 O = occipital rsEEG alpha 3 power density.*

| **Model** | **Predictors** | **β (± SE)** | **p-value** |
| --- | --- | --- | --- |
| **M12 Figure de Rey imm ~ Group * Edu * M12 A2 P**  F = 4.31, p < 0.05  R^2 = 0.172, Adjusted R^2 = 0.096 | Edu | 3.7411 ± 1.5682 | 0.0195 |
|  | M12 A2 P | 2.899 ± 1.2414 | 0.0222 |
|  | Group*Edu | -5.1266 ± 2.3179 | 0.03 |
|  | Edu*M12 A2 P | -6.0405 ± 2.0841 | 0.0049 |
|  | Group*Edu*M12 A2 P | 7.5272 ± 3.2654 | 0.0239 |
| **M12 DMS 48 1h score~ Group * Edu * M12 A2 O**  F = 6.98, p < 0.01  R^2 = 0.210, Adjusted R^2 = 0.137 | Group*Edu*M12 A2 O | -180.1224 ± 81.3533 | 0.0298 |
| **M12 Copy of the Rey figure immediate ~ Group * Edu * M12 A2 O**  F = 4.24, p < 0.05  R^2 = 0.159, Adjusted R^2 = 0.081 | Edu | 3.2096 ± 1.5286 | 0.0391 |
|  | Group*Edu | -5.0093 ± 2.3588 | 0.037 |
|  | Edu*M12 A2 O | -4.3499 ± 1.6909 | 0.012 |
|  | Group*Edu*M12 A2 O | 6.1846 ± 2.7938 | 0.0299 |
| **M12 Figure de Rey imm ~ Group * Edu * M12 A3 P**  F = 4.40, p < 0.05  R^2 = 0.189, Adjusted R^2 = 0.115 | Edu | 4.562 ± 1.7323 | 0.0102 |
|  | M12 A3 P | 3.5258 ± 1.3557 | 0.0112 |
|  | Group*Edu | -5.9207 ± 2.4075 | 0.0162 |
|  | Edu*M12 A3 P | -7.6505 ± 2.4456 | 0.0025 |
|  | Group*Edu*M12 A3 P | 9.0673 ± 3.4366 | 0.0101 |
| **M12 Copy of the Rey figure immediate ~ Group * Edu * M12 A3 O**  F = 4.36, p < 0.05  R^2 = 0.181, Adjusted R^2 = 0.105 | Edu | 3.9885 ± 1.6139 | 0.0157 |
|  | M12 A3 O | 2.2453 ± 1.0354 | 0.0332 |
|  | Group*Edu | -5.7691 ± 2.3531 | 0.0165 |
|  | Edu*M12 A3 O | -5.425 ± 1.8378 | 0.0042 |
|  | Group*Edu*M12 A3 O | 7.1833 ± 2.7642 | 0.0112 |

**Table SM5.** *Association between the rsEEG variables and the neuropsychological dependent variables at the M12 timepoint.* Results of a control statistical session based on linear regression models (p < 0.05 uncorrected), testing the effect of the Group (SMCneg and SMCpos) and Edu (Edu- and Edu+) predictors on the association between the rsEEG variables of interest and the neuropsychological dependent variables at the 12-month follow-up (M12) acquisition. The statistically significant results were observed for all the rsEEG power density variables considered, i.e., the posterior and occipital rsEEG alpha 2 and alpha 3 power density (p < 0.05 uncorrected). *Legend: M12 = 12-month follow-up acquisition; Copy of the Rey figure immediate = duration of the immediate copy of the Rey Figure (s);* *DMS 48 1h score = DMS 48 recognition after 1hour score; rsEEG = resting-state electroencephalographic; A2 P = parietal rsEEG alpha 2 power density; A2 O = occipital rsEEG alpha 2 power density; A3 P = parietal rsEEG alpha 3 power density; A3 O = occipital rsEEG alpha 3 power density.*

No statistically significant 3-way Group × Education × rsEEG variable interaction was observed at M24 (p > 0.05). The post-hoc analysis revealed that, only in the SMCpos Edu- sub-group, there was a statistically significant (p < 0.05, FDR corrected) negative association between the parietal and occipital alpha 2 and alpha 3 power density and the Copy of the Rey figure immediate (time) at M0 (Figure SM7) and M12 (Figure SM8). No statistically significant results were observed for M24.

**
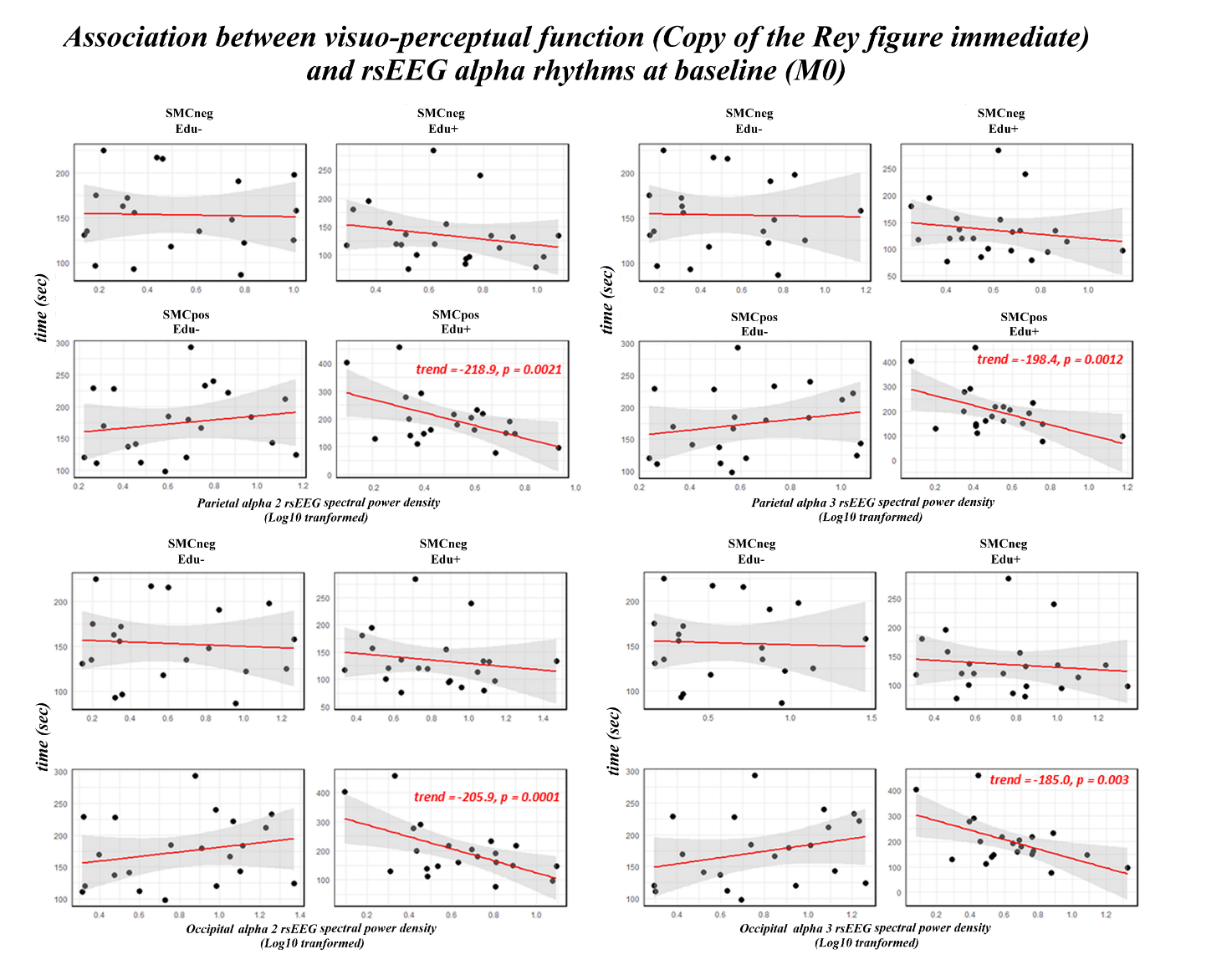
**

**Figure SM7.** *Association between the rsEEG variables and the neuropsychological dependent variables at the M0 timepoint.* Scatterplots of the Copy of the Rey figure immediate (time; estimated marginal means by General Linear Regression model) in the SMCneg-Edu- (upper left), SMCneg-Edu+ (upper right), SMCpos-Edu- (lower left), and SMCpos-Edu+ (lower right) sub-groups expressing the association with the parietal and occipital rsEEG alpha 2 (first column) and alpha 3 (second column) power density at the baseline (M0) acquisition. Inside each graph, the statistically significant associations calculated by the sub-group analysis using estimated marginal means are highlighted in red (p < 0.05; FDR corrected), as well as the slope coefficients and p-values. *Legend: SMC = subjective memory complaint negative (SMCneg) or positive (SMCpos) to amyloid load; M0 = baseline acquisition.*

**
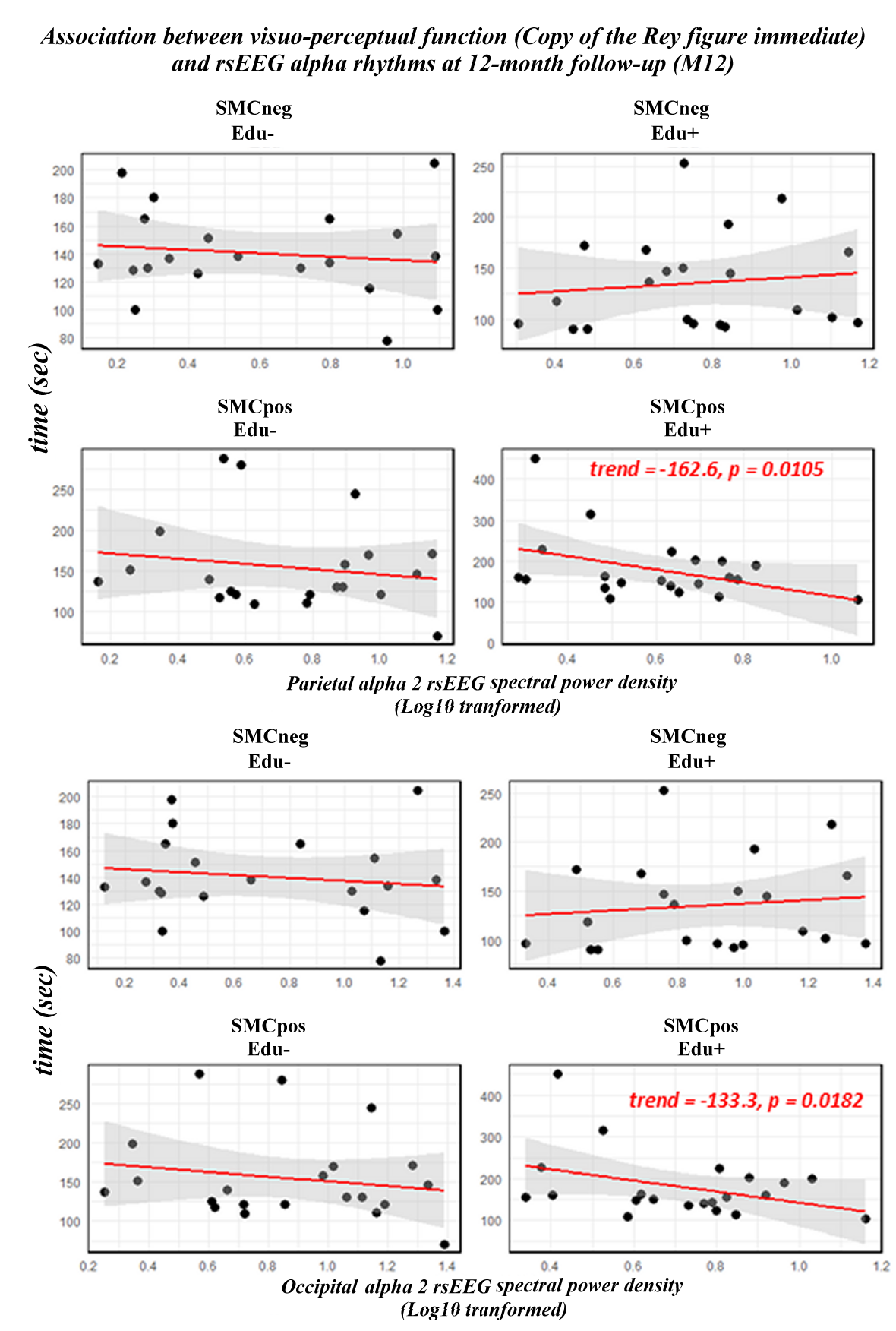
**

**Figure SM8.** *Association between the rsEEG variables and the neuropsychological dependent variables at the M12 timepoint.* Scatterplots of the Copy of the Rey figure immediate (time; estimated marginal means by General Linear Regression model) in the SMCneg-Edu- (upper left), SMCneg-Edu+ (upper right), SMCpos-Edu- (lower left), and SMCpos-Edu+ (lower right) sub-groups expressing the association with the parietal (upper) and occipital (lower) rsEEG alpha 2 power density at the 12-month follow-up (M12) acquisition. Inside each graph, the statistically significant associations calculated by the sub-group analysis using estimated marginal means are highlighted in red (p < 0.05; FDR corrected), as well as the slope coefficients and p-values. *Legend: SMC = subjective memory complaint negative (SMCneg) or positive (SMCpos) to amyloid load; M12 = 12-month follow-up acquisition.*

*2.6 Control analysis on the tauopathy in the SMCneg and SMCpos seniors*

In line with the hypothesis that amyloidosis is associated with and may drive the spreading of greater tau pathology, we evaluated the amyloid-beta (Aβ)1-42, phospho-tau and total-tau measures in the CSF in relation to the amyloidosis revealed by amyloid PET. Unfortunately, these data were available only for a minority of the participants enrolled in the present study. Due to the few existing cases, we performed an exploratory statistical analysis to compare the CSF measures in the SMCneg and SMCpos participants. To this aim, we performed several T-tests, having as dependent variables the CSF measure of Aβ1-42, total-tau, phospho-tau, ratio between the total-tau/Aβ1-42, ratio between the phospho-tau/Aβ1-42 an as grouping variable the Group (SMCneg, N = 7, SMCpos, N = 11). The statistical threshold of p < 0.05 was used.

The results of this analysis are illustrated in Figure SM9. For all the CSF variables analyzed, we observed a statistically significant difference between SMCneg and SMCpos seniors. More specifically, SMCpos seniors were characterized by lower Aβ1-42, higher total- and phospho-tau, as well as higher total-tau/Aβ1-42 and phospho-tau/Aβ1-42 in the CSF as compared to the SMCneg seniors (p < 0.05).

**
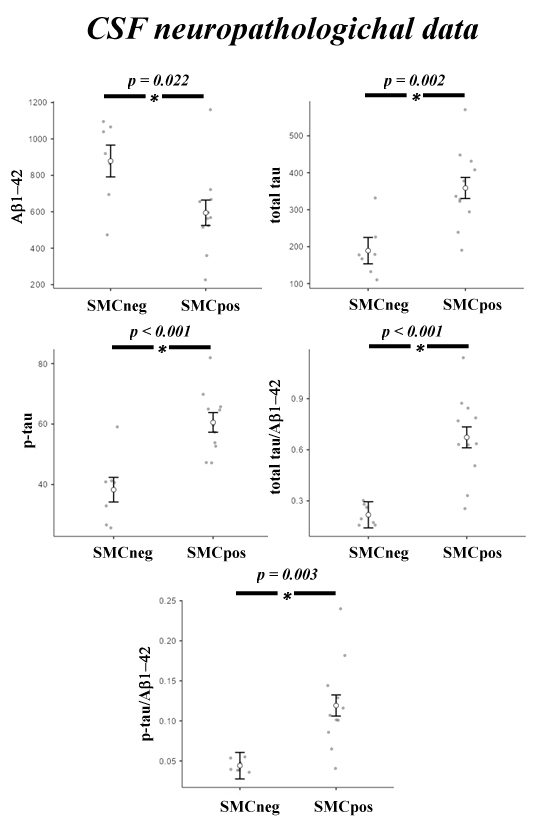
**

**Figure SM9.** *Cerebrospinal fluid (CSF)* *Aβ and tau in the SMCneg and SMCpos seniors.* Mean values (± standard error mean, SE) of the baseline CSF variables for the two groups of participants (SMCneg and SMCpos seniors). The t-tests showed a statistically significant difference between SMCneg and SMCpos seniors. More specifically, SMCpos seniors were characterized by lower Aβ1-42, higher total- and phospho-tau, as well as higher total-tau/Aβ1-42 and phospho-tau/Aβ1-42 as compared to the SMCneg seniors (p < 0.05). *Legend: SMC = subjective memory complaint negative (SMCneg) or positive (SMCpos) to amyloid load; Aβ = beta-amyloid.*

*2.7 Control analysis using educational attainment as a continuous variable*

We performed several regression analyses to confirm the main results of the rsEEG and MRI variables using continuous educational levels.

The first regression analysis used the posterior rsEEG alpha power density at the different timepoints (M0, M12, and M24) as the dependent variable and the educational attainment (as a continuous variable) and Group (SMCneg, SMCpos) as predictors:

- Dependent rsEEG variables: parietal and occipital rsEEG alpha 2 and alpha 3 power density at M0, M12, and M24 (one model for each variable).
- Predictors: Group (SMCneg, SMCpos), Education (continuous variable), and 2-way Group × Education interaction.

The second regression analysis used the neuroimaging measures at the different timepoints (M0 and M24) as the dependent variable and the educational attainment (as a continuous variable) and Group (SMCneg, SMCpos) as predictors.

- Dependent MRI variables: Alzheimer’s amyloid load measured by PET and MRI-based normalized grey matter volume, normalized white matter volume, normalized CSF volume, basal forebrain volume, hippocampus volume, entorhinal cortex volume, entorhinal cortex thickness, fusiform thickness, inferior parietal thickness, inferior temporal thickness, lateral occipital thickness, middle temporal thickness, para-hippocampal thickness, paracentral thickness, pericalcarine thickness, postcentral thickness, posterior cingulate thickness, precentral thickness, precuneus thickness, superior frontal thickness, superior parietal thickness, superior temporal thickness, insula thickness, white matter hyperintensities (one model for each variable).
- Predictors: Group (SMCneg, SMCpos), Education (continuous variable), and 2-way Group × Education interaction.

The model accuracy was assessed by evaluating the residual standard error (RSE) and adjusted R-squared (R^2). The residuals’ distribution was checked for heteroscedasticity, normality, and influential observations in the data (p < 0.05). The results are reported in Table SM6. There was a statistically significant 2-way Group × Education interaction (p < 0.05) in the association with the parietal and occipital rsEEG alpha 2 and alpha 3 power density at all the timepoints (M0, M12, M24). The post-hoc analysis revealed no statistically significant Group differences (p > 0.05, FDR corrected; Figure SM10). Concerning the MRI variables, only for the fusiform thickness at M0 and for the inferior parietal thickness at M24, there was a statistically significant 2-way Group × Education interaction (p < 0.05; Figure SM11).

| **Model** | **Predictors** | **β (± SE)** | **p-value** |
| --- | --- | --- | --- |
| **M0 A2 P ~ Group * educational attainment**  F = 4.72, p < 0.05  R^2 = 0.056, Adjusted R^2 = 0.027 | Group*educational attainment | -0.474 ± 0.028 | 0.0303 |
| **M0 A2 O ~ Group * educational attainment**  F = 6.63, p < 0.01  R^2 = 0.077, Adjusted R^2 = 0.042 | Group*educational attainment | -0.556 ± 0.035 | 0.012 |
| **M0 A3 O ~ Group * educational attainment**  F = 4.55, p < 0.05  R^2 = 0.056, Adjusted R^2 = 0.021 | Group*educational attainment | -0.466 ± 0.034 | 0.036 |
| **M12 A2 P ~ Group * educational attainment**  F = 8.48, p < 0.005  R^2 = 0.099, Adjusted R^2 = 0.066 | Group*educational attainment | -0.621 ± 0.028 | 0.005 |
| **M12 A2 O ~ Group * educational attainment**  F = 8.00, p < 0.01  R^2 = 0.091, Adjusted R^2 = 0.057 | Group*educational attainment | -0.597 ± 0.035 | 0.007 |
| **M12 A3 P ~ Group * educational attainment**  F = 5.78, p < 0.005  R^2 = 0.112, Adjusted R^2 = 0.079 | Group*educational attainment | -0.657 ± 0.027 | 0.003 |
| **M12 A3 P ~ Group * educational attainment**  F = 5.78, p < 0.005  R^2 = 0.112, Adjusted R^2 = 0.079 | Group*educational attainment | -0.657 ± 0.027 | 0.003 |
| **M24 A2 P ~ Group * educational attainment**  F = 4.35, p < 0.05  R^2 = 0.060, Adjusted R^2 = 0.030 | Group*educational attainment | -0.455 ± 0.023 | 0.040 |
| **M24 A2 O ~ Group * educational attainment**  F = 4.75, p < 0.05  R^2 = 0.062, Adjusted R^2 = 0.027 | Group*educational attainment | -0.476 ± 0.036 | 0.032 |
| **M0 fusiform thickness ~ Group * educational attainment**  F = 4.84, p < 0.05  R^2 = 0.113, Adjusted R^2 = 0.079 | Group*educational attainment | -0.473 ± 0.011 | 0.031 |
| **M24 inferior parietal thickness ~ Group * educational attainment**  F = 4.71, p < 0.05  R^2 = 0.138, Adjusted R^2 = 0.105 | Group*educational attainment | -0.460 ± 0.012 | 0.033 |

**Table SM6**. *Association between the educational attainment, the Group, and the rsEEG and MRI dependent variables of interest at the M0, M12, and M24 timepoint*. Results of a control statistical session based on linear regression models (p < 0.05 uncorrected), testing the effect of the Group (SMCneg and SMCpos) on the association between the educational attainment (continuous variable) and the rsEEG and MRI dependent variables of interest at the baseline (M0), 12-month (M12), and 24-month follow-up (M24) acquisition. The statistically significant results including the 2-way Group × education attainment interaction are reported (p < 0.05 uncorrected). *Legend: M0 = baseline acquisition; M12 = 12-month acquisition; M24 = 24-month follow-up acquisition; rsEEG = resting-state electroencephalographic; A2 P = parietal rsEEG alpha 2 power density; A2 O = occipital rsEEG alpha 2 power density; A3 P = parietal rsEEG alpha 3 power density; A3 O = occipital rsEEG alpha 3 power density.*

**
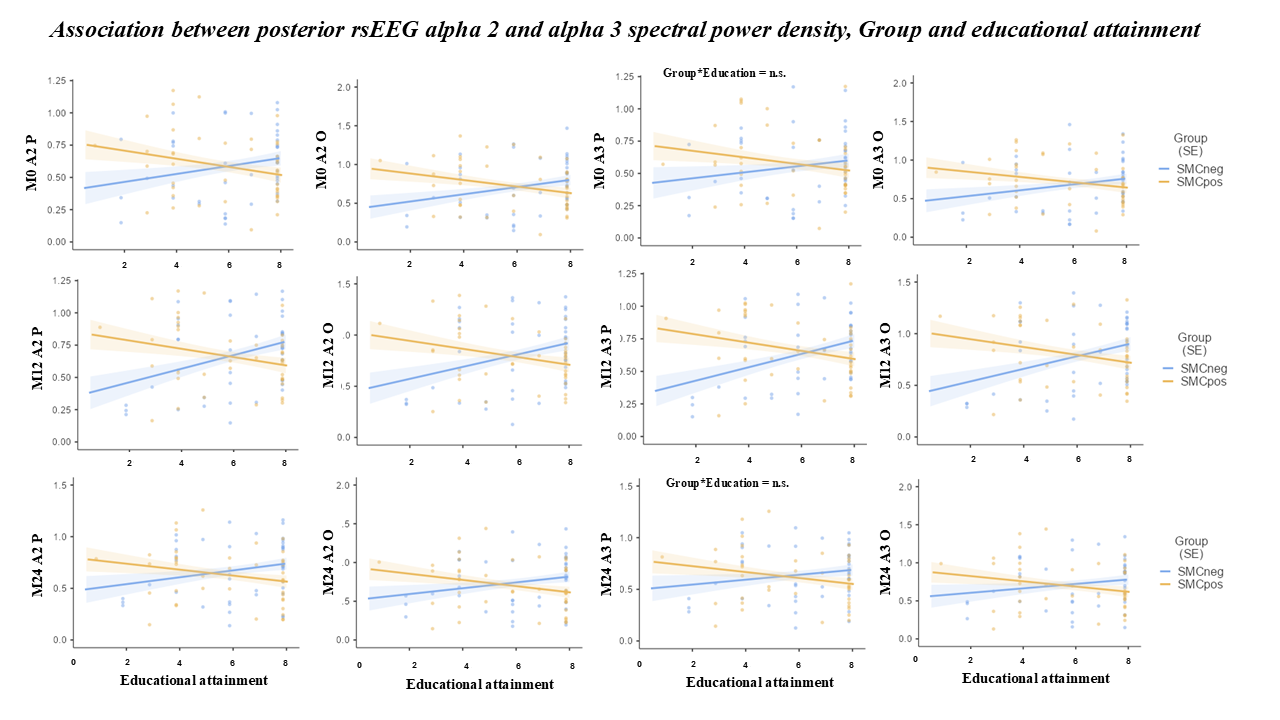
**

**Figure SM10.** *Plot of the association between the educational attainment, the Group, and the rsEEG dependent variables of interest at the M0, M12, and M24 timepoint*. Plots illustrate the estimated marginal means and the trends according to the Group (SMCneg, SMCpos) factor for the parietal and occipital rsEEG alpha 2 and alpha 3 power density. *Legend: M0 = baseline acquisition; M12 = 12-month acquisition; M24 = 24-month follow-up acquisition; SMC = subjective memory complaint negative (SMCneg) or positive (SMCpos) to amyloid load; rsEEG = resting-state electroencephalographic; A2 P = parietal rsEEG alpha 2 power density; A2 O = occipital rsEEG alpha 2 power density; A3 P = parietal rsEEG alpha 3 power density; A3 O = occipital rsEEG alpha 3 power density.*

***
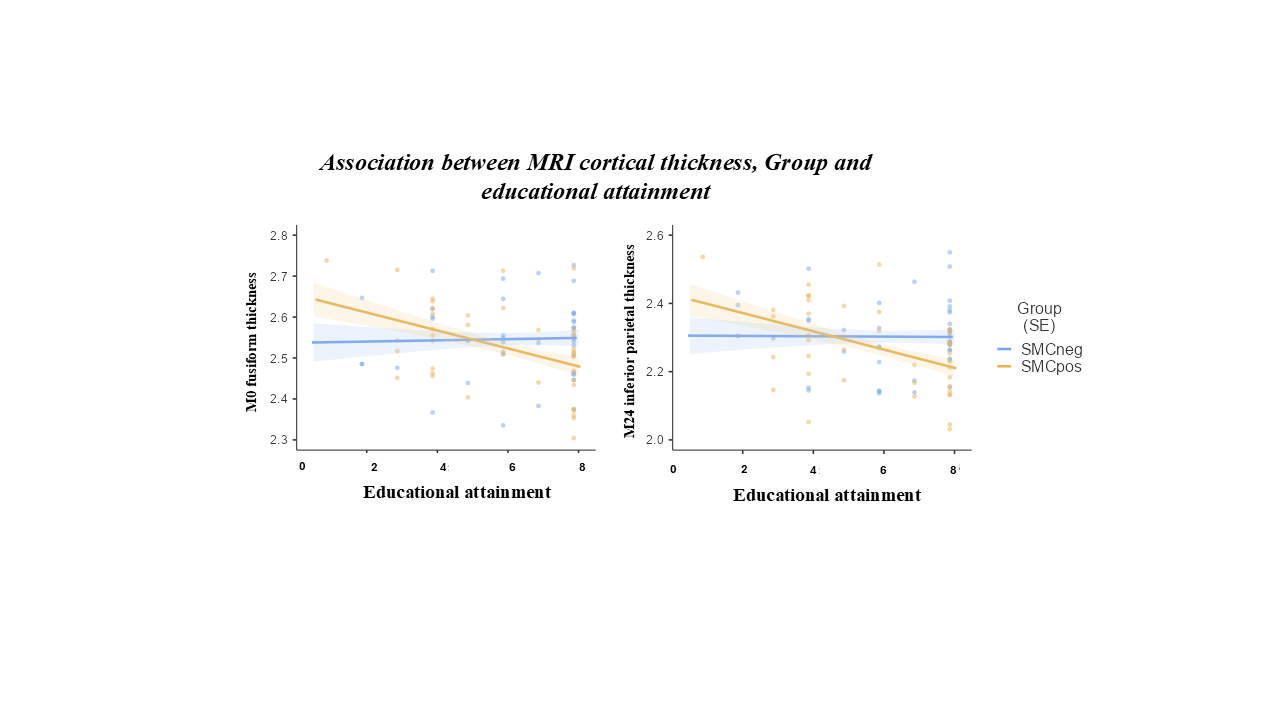
***

**Figure SM11*.*** *Plot of the association between the educational attainment, the Group, and the MRI dependent variables of interest at the M0 and M24 timepoint*. Plots illustrate the estimated marginal means and the trends according to the Group (SMCneg, SMCpos) factor for the fusiform (M0) and inferior parietal (M24) cortical thickness. *Legend: M0 = baseline acquisition; M24 = 24-month follow-up acquisition; SMC = subjective memory complaint negative (SMCneg) or positive (SMCpos) to amyloid load.*

*2.8 Control analysis on an adapted version of the short Cognitive Reserve Index questionnaire (CRIq)*

Educational attainment may be limited in its representativeness of CR, as it does not consider the duration and complexity of lifelong intellectual activities in work, cultural experience, and creative/leisure pursuits [3, 4]. Unfortunately, most of the above variables enriching CR were missing from the present INSIGHT-preAD database, such as the duration of the working activities, the qualification of the job complexity, and the specific details of the leisure activities (attending social events, theaters, conferences, etc.). To partially encompass the limited representativeness of educational attainment, we performed two control analyses based on a CR index enriched with additional information on working activity. To this purpose, we exploited the short Cognitive Reserve Index questionnaire (CRIq; [5]) as a conceptual reference. Specifically, we roughly estimated the duration of the working activities by subtracting the age of completion of educational studies (not lower than 18 years old) from the age at cessation of working activities. For the job qualification, we used the standard employee level for all the SMC cohort.

The first control analysis explored the association between educational attainment and relevant variables related to other CR dimensions, such as the lengthens of work period across the lifespan and leisure activities. Specifically, we computed the association between educational attainment and the following variables:

(i) *Age at cessation of work activity*. We hypothesized that higher educational attainment may predict a longer work period in the lifespan, given its association with intellectually demanding and interesting tasks that can motivate people to perform them in aging (e.g., problem-solving, decision-making, creativity) compared to simple, routine, and physical or manual labor activities.

(ii) *Satisfaction with actual life and frequency of interactions with neighbors and relatives*. We hypothesized that higher educational attainment may be associated with greater social interaction and engagement in leisure activities, as measured by self-reported life satisfaction and frequency of interactions with neighbors and relatives. Educational attainment may provide cognitive skills to take on the viewpoint of other people, anticipate their needs, and maintain social relationships. The data used for this control analysis were summarized in Table SM7.

|  | **SMCneg Edu-** | **SMCneg Edu+** | **SMCpos Edu-** | **SMCpos Edu+** | **Statistical comparison**  **(Edu- ≠ Edu+)** |
| --- | --- | --- | --- | --- | --- |
| **Age at cessation of**  **work activity** | 54.3 ± 2.4 | 60.4 ± 1.4 | 58.7 ± 1.3 | 62.3 ± 0.8 | **T-test:**  **Edu+ > Edu- =**  **p < 0.05** |
| **Satisfaction with actual life (0-4)** | 3.2 ± 0.1 | 3.0 ± 0.2 | 2.9 ± 0.1 | 3.0 ± 0.2 | T-test: n.s. |
| **Frequency of interactions with both neighbors and relatives**  **(0-10)** | 8.0 ± 0.4 | 8.2 ± 0.2 | 8.5 ± 0.2 | 8.3 ± 0.3 | T-test: n.s. |

**Table SM7**. Mean values (± standard error mean, SE) of the lengthens of work period across the lifespan and leisure activities indices for the SMC participants. *Legend: SMC = subjective memory complaint negative (SMCneg) or positive (SMCpos) to amyloid load.*

The main results of the first control analysis are reported in the following and Table SM8. A statistically significant difference between the Edu- and Edu+ sub-groups was observed in that the Edu+ sub-groups showed a mean older “Age at cessation of work activity” compared to the Edu- sub-groups (p < 0.05). No statistically significant differences between the Edu- and Edu+ sub-groups were observed for the other variables, such as “Satisfaction with actual life” and “Frequency of interactions with both neighbors and relatives.” Furthermore, only the main effect of the educational attainment was statistically significant (β = 1.68 ± 0.65 standard error mean, SE, p < 0.05) from a linear regression analysis with the educational attainment (continuous variable), the Group (SMCneg, SMCpos), and the 2-way interaction between them as predictors and “Age at cessation of work activity” as a target variable. These results are in line with the hypothesis that educational attainment is associated with features of the subsequent working activities relevant for the CR construct.

|  | **SMCneg CRI-** | **SMCneg CRI+** | **SMCpos CRI-** | **SMCpos CRI+** | **Statistical comparison**  **(CRI- ≠ CRI+)** |
| --- | --- | --- | --- | --- | --- |
| **N** | 20 | 22 | 20 | 22 | - |
| **Age** | 75.1 ± 0.6 | 75.6 ± 0.7 | 75.2 ± 0.8 | 76.7 ± 0.8 | T-test: n.s |
| **Sex (M/F)** | 4/18 (18%) | 11/9 (55%) | 6/16 (22%) | 9/11 (45%) | Fisher test: n.s. |
| **Education** | 4.9 ± 0.4 | 7.9 ± 0.1 | 4.3 ± 0.3 | 7.8 ± 0.1 | **T-test:**  **CRI+ > CRI- =**  **p < 0.05** |
| **MMSE** | 28.6 ± 0.2 | 28.9 ± 0.2 | 28.7 ± 0.2 | 28.9 ± 0.2 | Mann-Whitney U test: n.s |
| **APOE ε4**  **(Y/N)** | 6/16 (27%) | 3/17 (15%) | 10/12 (45%) | 4/16 (20%) | Fisher test: n.s |

**Table SM8.** Mean values (± standard error mean, SE) of the demographic, neuropsychological, and genetic data (presence or absence of at least one ε4 allele), together with the results of their statistical comparisons (p < 0.05) in the groups of seniors with subjective memory complaint (SMC) found to be amyloid amyloid-β negative (SMCneg) and positive (SMCpos) to the marker of Alzheimer’s neuropathology derived from 18F-florbetapir positron emission tomography (amyloid amyloid-β PET) stratified according to the low (CRI-) and high (CRI-) value of the adapted CRIq. *Legend: SMC = subjective memory complaint negative (SMCneg) or positive (SMCpos) to amyloid load; CRIq = Cognitive Reserve Index questionnaire [5]; MMSE = Mini-Mental State Examination; M/F = males/females; APOE = Apolipoprotein E; n.s. = not significant (p > 0.05).*

The second control analysis repeated some core analyses with a definition of CR using educational attainment combined with additional sources of information as a proxy for CR, such as rough estimates of the duration of working activities and the job qualification. The educational attainment, the estimated duration of the working activities, and job qualification were used to provide a rough CRI. The SMCneg and SMCpos participants were stratified according to the median CRI value within each group (see Table SM8 for the demographic and clinical features of the SMC sub-groups). With the SMC participants stratified based on the CRI, we used three ANOVA designs. The regional rsEEG power density, neuroimaging biomarkers (amyloid-β PET and structural MRI data), and the scores of cognitive-functional tests served as the dependent variables. The following results confirmed and cross-validated those of the main analyses grounded on the educational attainment only to stratify the SMC participants.

In the first ANOVA design, we used the regional rsEEG power density as a dependent variable and the Time (M0, M12, M24), Group (SMCneg and SMCpos), CRI (CRI- and CRI +), Band (delta, theta, alpha 1, alpha 2, alpha 3, beta 1, beta 2, and gamma), and ROI (frontal, central, parietal, occipital, and temporal) as factors. A statistically significant 4-way Group × CRI × Band × ROI interaction was observed (F(28, 2212) = 7.8476, p < 0.0001, gender as covariate). Duncan’s post-hoc analysis (p < 0.0025, equivalent to p < 0.05 Bonferroni corrected) confirmed the findings of the main analysis. Compared with the SMCneg CRI- sub-group, the SMCneg CRI+ sub-group exhibited higher parietal (p < 0.0001) and occipital (p < 0.0001) rsEEG alpha 2 power density as well as higher parietal (p < 0.0001) and occipital (p < 0.0002) rsEEG alpha 3 power density, in line with the “neuroprotective” hypothesis. Opposite effects of the CRI were observed in the SMCpos seniors. Compared with the SMCpos CRI- sub-group, the SMCpos CRI+ sub-group exhibited lower parietal (p < 0.00001) and occipital (p < 0.0005) rsEEG alpha 2 power density as well as lower parietal (p < 0.0001) and occipital (p < 0.000005) rsEEG alpha 3 power density, in line with the “compensatory” hypothesis (Figure SM12).

**
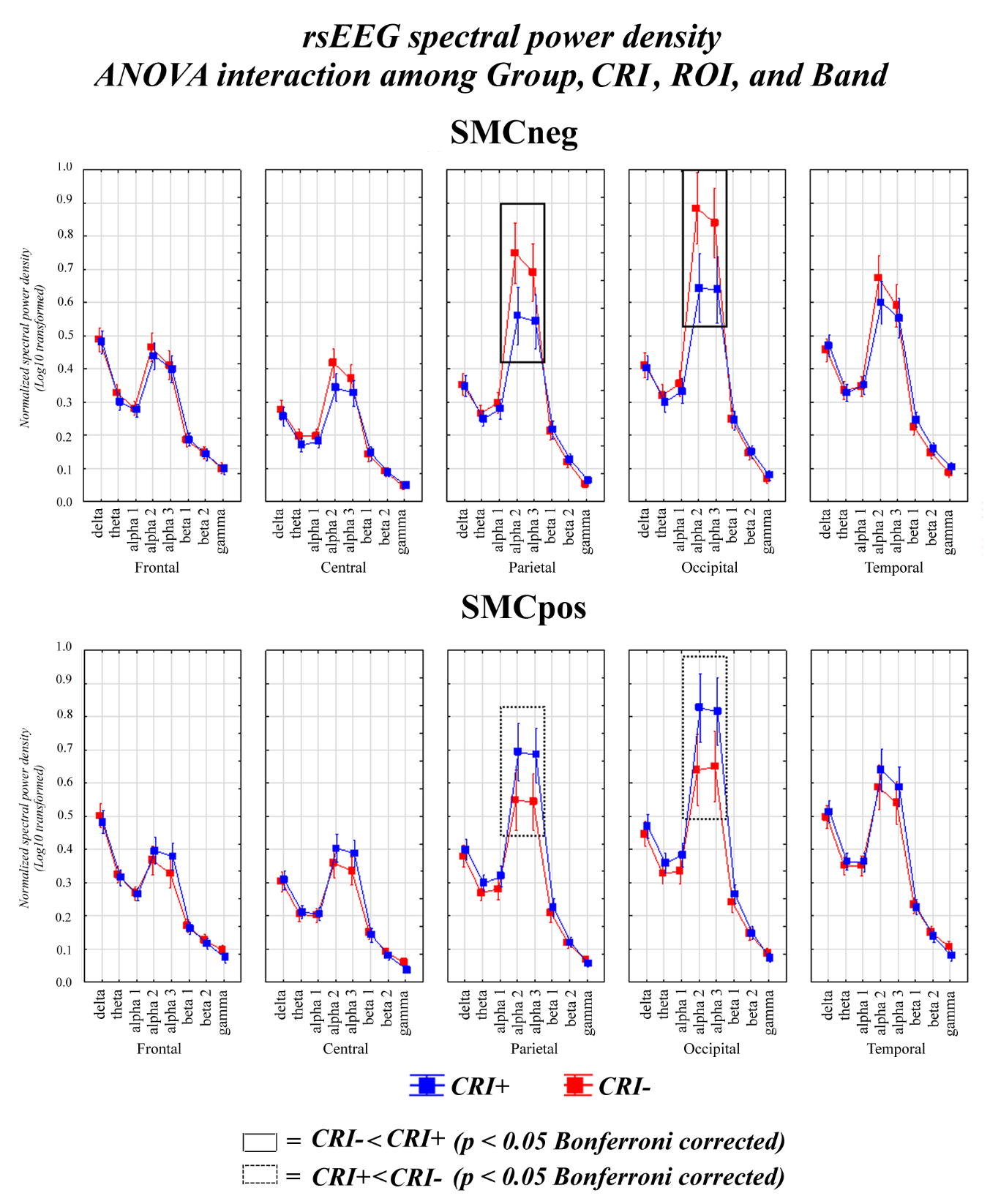
**

**Figure SM12.** *Effect of Cognitive Reserve Index (CRI) on the rsEEG rhythms in the SMCneg and SMCpos seniors.* Mean values (± standard error mean, SE) of normalized rsEEG power density for (1) two groups (SMCneg and SMCpos seniors), (2) two CRI levels (CRI- and CRI+), (3) five ROIs (frontal, central, parietal, occipital, and temporal), and (4) eight frequency bands (delta, theta, alpha 1, alpha 2, alpha 3, beta 1, beta 2, and gamma). The ANOVA showed a statistically significant 3-way Group × CRI × Band × ROI interaction (F(28, 2212) = 7.85, p < 0.0001, gender as covariate). The rectangles indicate the scalp regions and frequency bands in which the rsEEG power density presented a statistically significant pattern: CRI+ ≠ , CRI- in the SMCneg and SMCpos senior groups (Duncan post hoc test, p < 0.05 corrected for multiple comparisons = p < 0.0025; see “Methods” for the criterion of such correction). CRI was estimated by adapting the short version of the CRI questionnaire (CRIq; [5]). *Legend: SMC = subjective memory complaint negative (SMCneg) or positive (SMCpos) to amyloid load; rsEEG = resting-state electroencephalographic.*

In the second ANOVA design, we used the neuroimaging variables (amyloid-β PET and structural MRI data) as the dependent variables in several ANOVAs having Time (M0, M24), Group (SMCneg, SMCpos), and CRI (CRI-, CRI+) as factors. A statistically significant 2-way Group × CRI interaction was observed for the inferior parietal (F (1, 78) = 6.57, p < 0.01, gender as covariate) and middle temporal thickness computed from MRIs (F (1, 78) = 1.579, p < 0.05, gender as covariate). The Duncan planned post-hoc test (p < 0.0125, equivalent to p < 0.05 corrected) showed no differences between the SMCneg sub-groups. Compared with the SMCpos Edu- sub-group, the SMCpos Edu+ sub-group exhibited lower inferior parietal thickness in line with the “compensatory” hypothesis (p < 0.00125) both at M0 and M24. No statistically significant post-hoc differences were observed for the middle temporal thickness (Figure SM13).

**
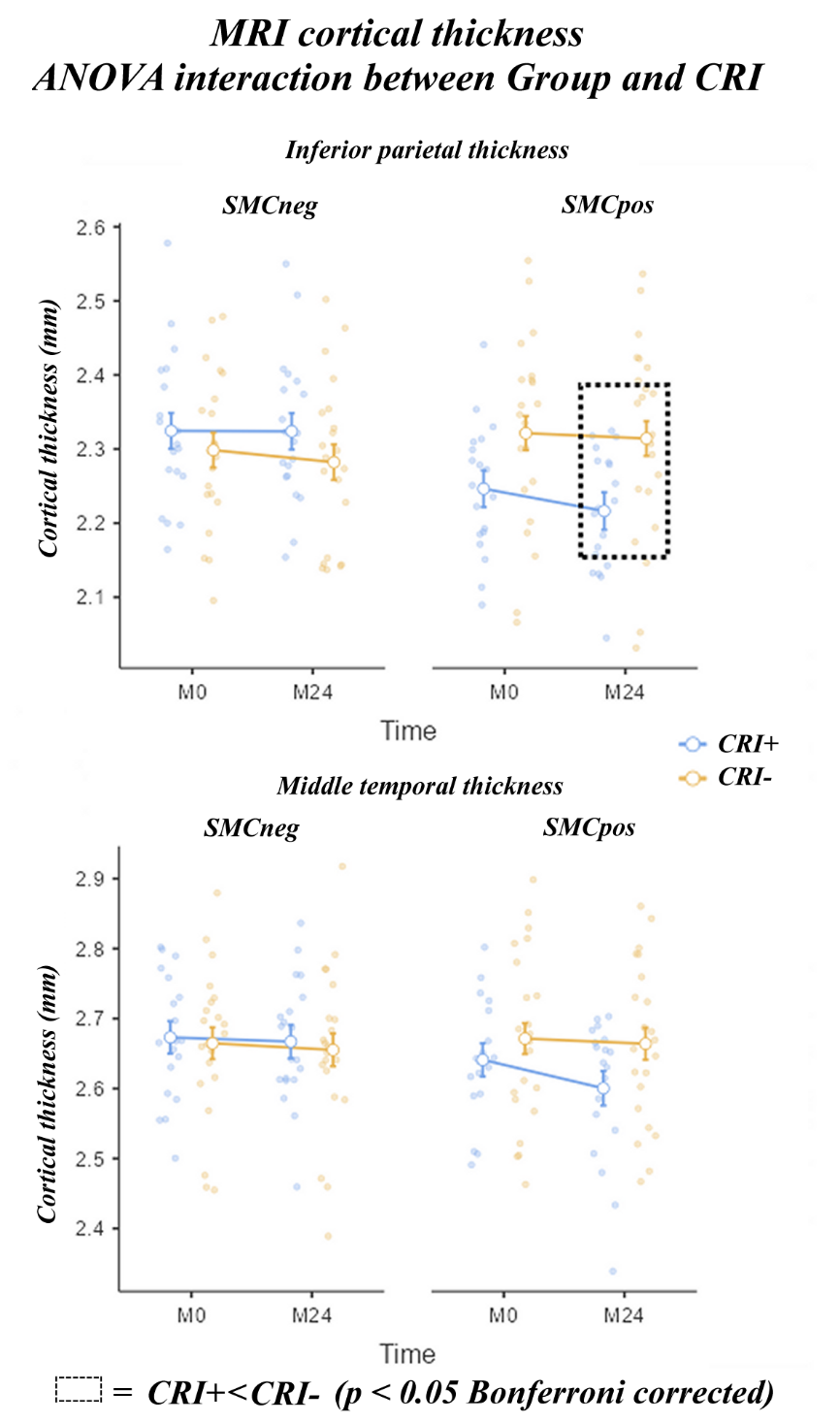
**

**Figure SM13*.*** *Effect of Cognitive Reserve Index (CRI) on the MRI variables in the SMCneg and SMCpos seniors.* Mean values (± standard error mean, SE) of the cortical inferior parietal thickness for: (1) two groups (SMCneg and SMCpos seniors), (2) two Education levels (Edu+ and Edu-), and (4) two timepoints, namely the baseline (M0) and 24-month follow-up (M24). No statistically significant effect was produced by the ANOVA design (p > 0.05) using the factors Time, Group, and Edu. The ANOVA showed a statistically significant 2-way Group × CRI interaction effect (F (1, 78) = 6.57, p < 0.01, gender as covariate). Only for the SMCpos seniors, a statistically significant difference CRI+ ≠ CRI- was observed both at the M0 and M24 timepoints for the inferior parietal thickness (Duncan post hoc test, p < 0.05 corrected for multiple comparisons = p < 0.0125; see “Methods” for the criterion of such correction). No statistically significant effects were observed for the middle temporal thickness. CRI was estimated by adapting the short version of the CRI questionnaire (CRIq; [5]). *Legend: SMC = subjective memory complaint negative (SMCneg) or positive (SMCpos) to amyloid load; MRI = magnetic resonance imaging.*

In the third ANOVA design, we used the scores of the cognitive-functional tests as dependent variables in several ANOVAs having Time (M0, M24), Group (SMCneg, SMCpos), and CRI (CRI-, CRI+) as factors. No statistically significant 2-way Group × CRI interaction effect was observed (p > 0.05).

**3. *Supplementary Discussion***

*3.1 Methodological remarks*

The following methodological limitations should be considered when interpreting the results of the present study.

This retrospective study was based on data collected by a single French clinical unit. On the one hand, this ensured the maximum methodological consistency during the study development. On the other hand, the results require a cross-validation from a multicentric study with independent and "harmonized" clinical units should be performed.

The relatively small number of SMC participants in each sub-group of the study limits the generalizability of the present findings, making them susceptible to interpretation and needing confirmation in larger cohorts.

As mentioned above, educational attainment may only partially reflect CR, as it primarily captures early-life intellectual development and does not formally account for the duration and complexity of lifelong cognitive engagement through occupational, cultural, and leisure activities. Within this framework, occupational complexity has gained attention in the study of neurodegenerative diseases, as it may contribute to resilience against the cognitive effects of Alzheimer’s disease (AD) pathology (e.g., see [3]). Recent studies have highlighted that occupational complexity mediates a substantial portion (e.g., up to 73%) of the association between education and dementia, even when controlling health-related confounders [4]. Unfortunately, we do not have this information available in the INSIGHT-preAD database. For this reason, we estimated a very rough CRI [5] with surrogates of duration and quality of the working activities. Afterward, we stratified SMCneg and SMCpos participants according to the median CRI value within each group and repeated the main statistical analyses. These analyses included ANOVAs on rsEEG power density, neuroimaging measures (amyloid-β PET and structural MRI), and cognitive-functional performances as assessed by neuropsychological testing. We tested the consistency with the findings of the main analyses. The results of the control analyses confirmed that educational attainment represents an important aspect of the CR construct. Furthermore, the results based on the enriched CR construct were consistent with those obtained using educational attainment only. Overall, the results based on the enriched CR construct replicated the “neuroprotective” and “compensatory” effects on rsEEG and structural MRI measures in the SMCneg and SMCpos groups, respectively. Finally, there were no significant changes in rsEEG and structural MRI measures at the follow-ups, as in the results of the main data analysis.

Due to the intensive experimental design, only one minute of eyes-closed rsEEG recordings were available, affecting the reliability of power density estimation. In our study we did not observe any effect of amyloid and cognitive reserve on other frequency bands beyond alpha rhythms, including the theta and gamma frequency that were previously acknowledged to predict amyloid positivity in preclinical research [6, 7]. Focusing on the theta-alpha subtypes alterations, we did not observe any interacting or main effect of the educational attainment and amyloid load on the transition frequency (TF) and individual alpha frequency peak (IAFp) along time, suggesting no evidence of rsEEG alpha slowing over the 24-month follow-up period. Gamma activity increase has been related to AD neuropathology [8], ranging between approximately 30 and 80 Hz. Our recordings lacked the frequency dynamics and resolution necessary to capture such alterations, especially given that background gamma activity during rsEEG is less prominent than slower rhythms. Future studies should assess whether gamma alterations may be sensitive to preclinical stages and to early progression along AD. Our approach also did not capture higher-order cross-frequency theta-gamma dynamics, such as the cross-frequency coupling (CFC), typically assessing the modulation of gamma oscillatory amplitude by the theta phase (phase-amplitude CFC; [6, 7]). This phenomenon is particularly involved in the working memory processing, as revealed by event-related paradigms. Future research should evaluate its relationship with preclinical AD stages.

A relatively short observation period (24 months) was used. As the disease evolves over decades with subtle pathophysiological and clinical manifestations, it is recommended to follow the participants for longer periods of time to observe these changes.

The limited effect observed may be due to an underestimation of the complex interactions between Aβ, tau and other co-factors. Due to the very small number of cases in which tauopathy was quantified, we cannot draw specific or conclusive evidence on its association with CR-related mechanisms. Tauopathy is more directly associated with neurodegeneration than amyloidosis [9], whereas Aβ-tau synergy is associated with brain dysfunction [10-12], atrophy [13], and cognitive decline [14, 15]. Recent research highlights additional contributors to the biological heterogeneity of AD, including comorbidities (e.g., cardiovascular), neuroinflammation, and genetic risk factors [16]. The pathophysiological stage in cognitively healthy individuals varies significantly in the preclinical phase, complicating the establishment of a clear link between Aβ accumulation and cognitive trajectories, both of which follow nonlinear progression patterns throughout AD [17].

**4. *References***

1. Barbeau E, Didic M, Tramoni E, et al. Evaluation of visual recognition memory in MCI patients. Neurology. 2004;62(8):1317-1322. doi:10.1212/01.wnl.0000120548.24298.db
2. Rey A, Osterrieth PA. (1941). Rey-Osterrieth Complex Figure Copying Test. APA PsycTests. <https://doi.org/10.1037/t07717-000>
3. Boots EA, Schultz SA, Almeida RP, et al. Occupational Complexity and Cognitive Reserve in a Middle-Aged Cohort at Risk for Alzheimer's Disease. Arch Clin Neuropsychol. 2015;30(7):634-642. doi:10.1093/arclin/acv041
4. Taylor K, Marston L, Mukadam N. Mediation of the association between education and dementia by occupational complexity, income, health behaviours and health outcomes. BMC Psychiatry. 2025;25(1):174. Published 2025 Feb 25. doi:10.1186/s12888-025-06619-4
5. Mondini S, Pucci V, Pastore M, Gaggi O, Tricomi PP, Nucci M. s-CRIq: the online short version of the Cognitive Reserve Index Questionnaire. Aging Clin Exp Res. 2023;35(12):2903-2910. doi:10.1007/s40520-023-02561-1
6. Mehak SF, Shivakumar AB, Kumari S, Muralidharan B, Gangadharan G. Theta and gamma oscillatory dynamics in mouse models of Alzheimer's disease: A path to prospective therapeutic intervention. Neurosci Biobehav Rev. 2022;136:104628. doi:10.1016/j.neubiorev.2022.104628.
7. Calvin-Dunn KN, Mcneela A, Leisgang Osse A, et al. Electrophysiological insights into Alzheimer's disease: A review of human and animal studies. Neurosci Biobehav Rev. 2025;169:105987. doi:10.1016/j.neubiorev.2024.105987
8. Wang J, Fang Y, Wang X, Yang H, Yu X, Wang H. Enhanced Gamma Activity and Cross-Frequency Interaction of Resting-State Electroencephalographic Oscillations in Patients with Alzheimer's Disease. Front Aging Neurosci. 2017;9:243. Published 2017 Jul 26. doi:10.3389/fnagi.2017.00243
9. Jack CR Jr, Andrews SJ, Beach TG, et al. Revised criteria for the diagnosis and staging of Alzheimer's disease. Nat Med. 2024;30(8):2121-2124. doi:10.1038/s41591-024-02988-7
10. Klunk WE, Engler H, Nordberg A, et al. Imaging the pathology of Alzheimer's disease: amyloid-imaging with positron emission tomography. *Neuroimaging Clin N Am*. 2003;13(4):781-ix. doi:10.1016/s1052-5149(03)00092-3
11. Hanseeuw BJ, Betensky RA, Jacobs HIL, et al. Association of Amyloid and Tau With Cognition in Preclinical Alzheimer Disease: A Longitudinal Study [published correction appears in JAMA Neurol. 2019 Aug 1;76(8):986. doi: 10.1001/jamaneurol.2019.2144]. *JAMA Neurol*. 2019;76(8):915-924. doi:10.1001/jamaneurol.2019.1424
12. Morris JC, Roe CM, Grant EA, et al. Pittsburgh compound B imaging and prediction of progression from cognitive normality to symptomatic Alzheimer disease. *Arch Neurol*. 2009;66(12):1469-1475. doi:10.1001/archneurol.2009.269
13. Gordon BA, McCullough A, Mishra S, et al. Cross-sectional and longitudinal atrophy is preferentially associated with tau rather than amyloid β positron emission tomography pathology. *Alzheimers Dement (Amst)*. 2018;10:245-252. Published 2018 Mar 6. doi:10.1016/j.dadm.2018.02.003
14. Hanseeuw B, Jacobs HIL, Schultz AP, et al. Longitudinal hippocampal atrophy is associated with an amyloid-independent entorhinal tauopathy and an amyloid-dependent neocortical tauopathy. *Alzheimer’s & Dementia*. 2020;16(S5):e045733. [doi:10.1002/alz.045733](https://doi.org/10.1002/alz.045733)
15. Shi J, Sabbagh MN, Vellas B. Alzheimer's disease beyond amyloid: strategies for future therapeutic interventions. *BMJ*. 2020;371:m3684. Published 2020 Oct 9. doi:10.1136/bmj.m3684
16. Ritchie K, Carrière I, Berr C, et al. The clinical picture of Alzheimer's disease in the decade before diagnosis: clinical and biomarker trajectories. *J Clin Psychiatry*. 2016;77(3):e305-e311. doi:10.4088/JCP.15m09989
17. Zhuo J, Zhang Y, Liu Y, et al. New Trajectory of Clinical and Biomarker Changes in Sporadic Alzheimer's Disease. *Cereb Cortex*. 2021;31(7):3363-3373. doi:10.1093/cercor/bhab017
